# Material discovery by combining stochastic surface walking global optimization with a neural network†
†Electronic supplementary information (ESI) available: Derivation for the gradient of *J*_σ_ with respect to NN parameters. DFT calculation setups. Parameters of atom-centered symmetry functions for generating TiO_2_ NN potential. Comparison of the performance of symmetry functions with different numbers of cutoff radii. Comparison of the computational cost of NN and DFT calculations. Comparison of structural properties of experimentally known TiO_2_ phase using NN and DFT PES. *XYZ* coordination of structures. See DOI: 10.1039/c7sc01459g



**DOI:** 10.1039/c7sc01459g

**Published:** 2017-06-30

**Authors:** Si-Da Huang, Cheng Shang, Xiao-Jie Zhang, Zhi-Pan Liu

**Affiliations:** a Collaborative Innovation Center of Chemistry for Energy Material , Key Laboratory of Computational Physical Science (Ministry of Education) , Shanghai Key Laboratory of Molecular Catalysis and Innovative Materials , Department of Chemistry , Fudan University , Shanghai 200433 , China . Email: zpliu@fudan.edu.cn

## Abstract

While the underlying potential energy surface (PES) determines the structure and other properties of a material, it has been frustrating to predict new materials from theory even with the advent of supercomputing facilities. The accuracy of the PES and the efficiency of PES sampling are two major bottlenecks, not least because of the great complexity of the material PES. This work introduces a “Global-to-Global” approach for material discovery by combining for the first time a global optimization method with neural network (NN) techniques. The novel global optimization method, named the stochastic surface walking (SSW) method, is carried out massively in parallel for generating a global training data set, the fitting of which by the atom-centered NN produces a multi-dimensional global PES; the subsequent SSW exploration of large systems with the analytical NN PES can provide key information on the thermodynamics and kinetics stability of unknown phases identified from global PESs. We describe in detail the current implementation of the SSW-NN method with particular focuses on the size of the global data set and the simultaneous energy/force/stress NN training procedure. An important functional material, TiO_2_, is utilized as an example to demonstrate the automated global data set generation, the improved NN training procedure and the application in material discovery. Two new TiO_2_ porous crystal structures are identified, which have similar thermodynamics stability to the common TiO_2_ rutile phase and the kinetics stability for one of them is further proved from SSW pathway sampling. As a general tool for material simulation, the SSW-NN method provides an efficient and predictive platform for large-scale computational material screening.

## Abstract 2

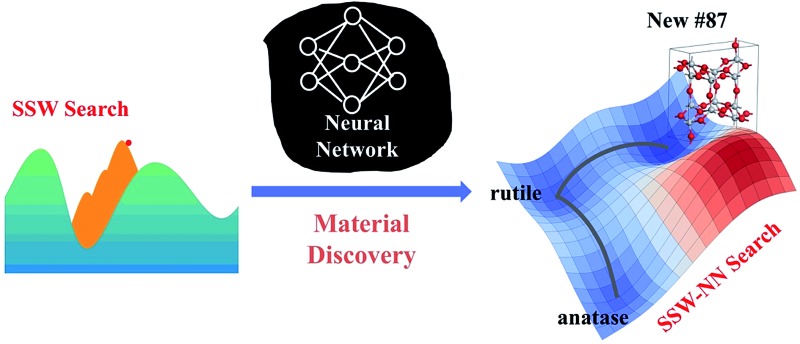
A powerful material discovery tool is invented by combining SSW global optimization with neural network computing, which identifies unprecedented TiO_2_ phases.

## 1. Introduction

Material discovery is a main theme in chemistry. Compared to synthetic experiments, finding new materials from theory has long been formidable: the accurate global potential energy surface (PES) and efficient PES sampling are two key prerequisites in order to screen out desirable structures from a large data set of PESs. For the past few decades, great advances have been achieved: (i) large scale density-functional theory (DFT) calculations emerged as a reliable and convenient tool for PES construction;^1^ (ii) advanced PES sampling techniques^2–12^ were developed to speed up the global PES exploration in order to find structures and pathways. However, the high computational demand intrinsic to electronic structure calculations severely restricts its application in systems with complex PESs (*e.g.* with more than 100 atoms), where the proper sampling of PESs takes excessively long computational times. The current dilemma in material discovery thus called for a breakthrough in methodology that should achieve accurate global PESs, *e.g.* at the level of DFT calculations, and should be suitable for efficient global structure and pathway prediction.

In the past 20 years, neural network (NN) function fitting techniques have demonstrated great potential for constructing high-dimensional PESs with a high accuracy.^13–16^ For example, these techniques have been successfully utilized for predicting the reaction dynamics of small molecules^17^ (*e.g.* less than 15 degrees of freedom) by fitting the PES data from high level quantum mechanics calculations. Recent advances in NN PES construction may be divided into two major streams: developing better NN architecture and improving NN training efficiency.^13,14,18–23^ Belonging to the former are, for example, the atom-centered high dimensional NN scheme^13,24^ introduced by Behler and Parinello to decompose total energy into individual atomic energies, and more recently the development of deep NNs^22^ by increasing the number of nonlinear layers to add more flexibility to the network. The state-of-the-art training method now simultaneously fits energy and forces,^21^ and utilizes quasi-Newton optimization methods (*e.g.* global extended Kalman filter^18,19^ and the Levenberg–Marquardt method^20^) to learn network parameters. While the NN-based PES is analytic and its accuracy is in principle comparable to quantum mechanics calculations (by which the training data set is obtained), the application of the method for material discovery has been rare due to some inherent drawbacks of NNs. In particular, the poor predictive ability is noticed for structures that are vastly different from the training data set.

The predictive power of NN PESs can be systematically improved by expanding the training set to incorporate as many structures as possible with different chemical environments, *e.g.* solids, surfaces and clusters. In practice, these structures may be randomly generated or obtained from PES sampling techniques, such as molecular dynamics, hybrid Monte Carlo,^25,26^ and metadynamics.^5,6^ Even so, a self-consistent iterative procedure by repeating data-expanding and NN-retraining is inevitable to ensure the robustness for the results from NN. In practical applications, this tedious and time-consuming procedure is a main obstacle in constructing global NN PESs and thus largely prevents wide applications in material discovery. Therefore, an automated and simple solution to generate a big training set representative for global PESs becomes an increasingly important challenge in the field.

In this work, we proposed a “Global-to-Global” approach for constructing the general-purpose NN PES for material discovery. The first “Global” means that we rely solely on our recently-developed stochastic surface walking (SSW) global optimization method^11,12^ to generate the training set based on quantum mechanics calculations, which explores exhaustively the PESs of relatively small systems (with periodic boundary conditions); the second “Global” indicates that the obtained NN PES is consistent with the quantum mechanics PES in a wide energy window, and thus functions properly in practice for the global optimizations of large systems, where the total energy per formula unit (f.u.) often varies by more than 10 eV. We will show that the utilization of the SSW global optimization training set largely eliminates the lengthy iterative NN improving procedure, and more importantly, renders the high transferability of the NN PES that is ideal for material discovery.

This article is organized as follows. In Section 2, we present the SSW-NN method to achieve the “Global-to-Global” goal, including the NN construction, the training method and the automated global training set generation based on the SSW method. In Section 3, we apply the SSW-NN method to an important material, namely TiO_2_, and benchmark the overall performance of the NN PES against quantum mechanics calculations. In Section 4, we make the first attempt to use the NN PES in the global optimization of large TiO_2_ systems. We successfully identify two unprecedented hollow-cage TiO_2_ crystal structures that are as stable as common TiO_2_ rutile phase. The implications of this work for material discovery are then discussed.

## 2. SSW-NN method

### a. PES fitting by neural network

#### Neural network architecture

**Fig. 1:**
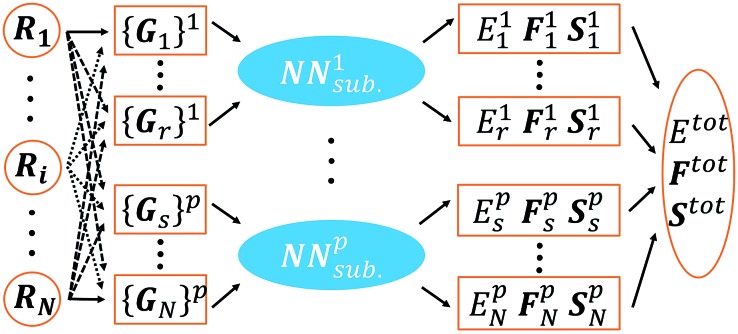
Scheme for our implementation of the Behler–Parinello-type NN. The meanings of the super/subscripts are as follows: *i*, *r*, *s*: atom indices; *N*: total number of atoms in a structure; *p*: element type index. The NN contains separated element-based subnets, NN_sub._^*p*^, where the inputs are a set of atom-centered symmetry functions {*G*_*i*_}^*p*^ (belonging to the *i* atom with the element type *p*) constructed from Cartesian coordinates {*R*} of a structure, and the outputs are the atomic properties {*E*_*i*_^*p*^, *F*_*i*_^*p*^, *S*_*i*_^*p*^}, *i.e.* energy, forces and stresses. The overall properties, *E* ^tot^, *F* ^tot^ and *S* ^tot^, can be calculated from the individual atomic contributions.

In this work we utilize the high dimensional neural network (HDNN) scheme introduced by Behler and Parinello to construct the NN. The NN architecture is schematically shown in Fig. 1. Since the HDNN scheme has been extensively reviewed previously,^15^ here, for the flow and clarity of the paper, we summarize briefly the key assumption of the approach, and highlight the algorithm for generating NN input nodes, the so-called atom-centered symmetry functions (ACSFs).

In the HDNN scheme, the total energy *E* ^tot^ of a structure can be decomposed and written as a linear combination of atomic energy *E*_*i*_ (eqn (1)), an assumption widely used in empirical force field calculations.1
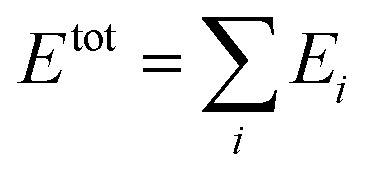

*E*_*i*_ for the atom *i* with the element type *p* can be obtained as the output from the standard neural network for this element, *i.e.* an element-based subnet NN_sub._^*p*^ (see Fig. 1). The input nodes for each subnet are a set of geometry-based symmetry functions {***G***_*i*_}^*p*^, as detailed in eqn (2)–(7), which are the functions of internuclear distances *R*_*ij*_ and angles *θ*_*ijk*_ = *a* cos(***R***_*ij*_·***R***_*ik*_/*R*_*ij*_·*R*_*ik*_) (*i*, *j*, *k* are atom indices). These ACSFs are translational and rotational invariant, and are able to depict the surrounding chemical environment of the atom with the adjustable parameters *R*_c_, *R*_s_, *η*, *κ*, *ζ*, and *λ*. More explanation on ACSF can be found in ref. 24.2
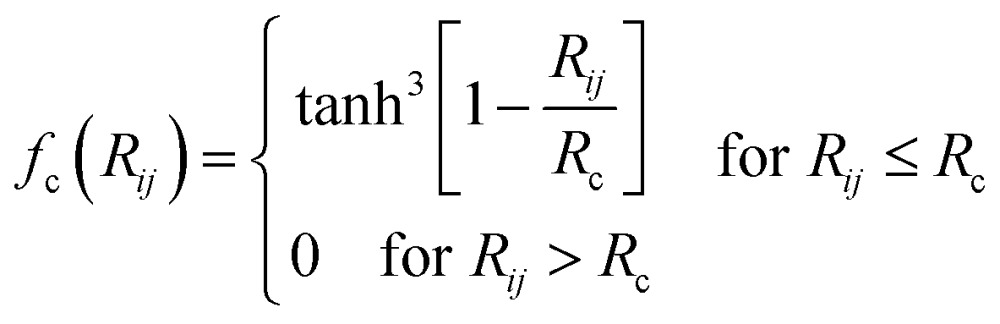

3
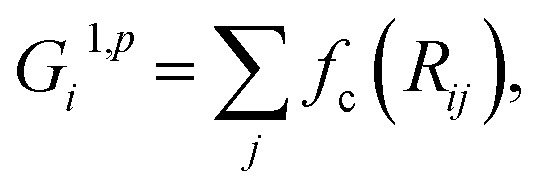

4
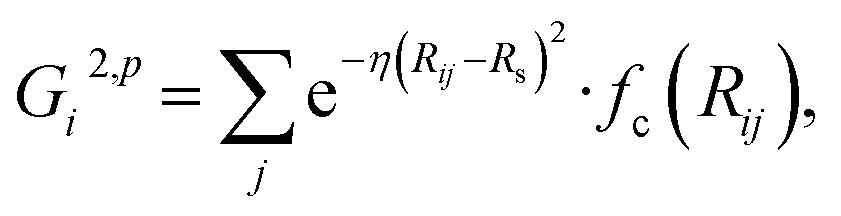

5
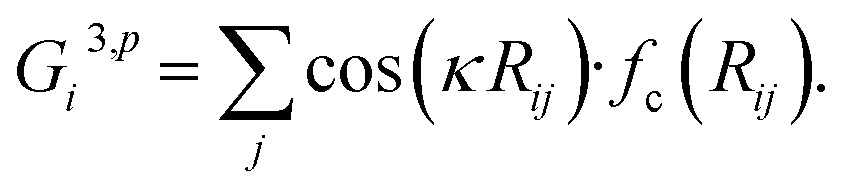

6
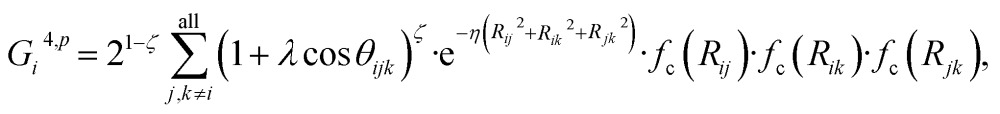

7
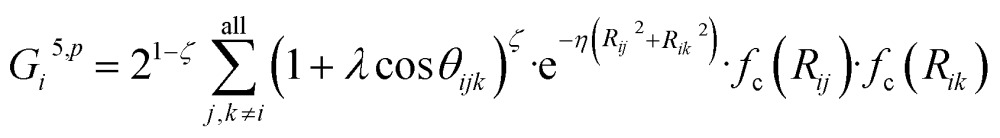



The atomic force can be analytically derived according to eqn (8), where the force component *F*_*k*,*α*_, *α* = *x*, *y* or *z*, acting on the atom *k* is the derivative of the total energy with respect to its coordinate *R*_*k*,*α*_. By combining with eqn (1), the force component can be further related to the derivatives of the atomic energy with respect to *j* ^th^ symmetry functions of atom *i*, *G*_*j*,*i*_:8
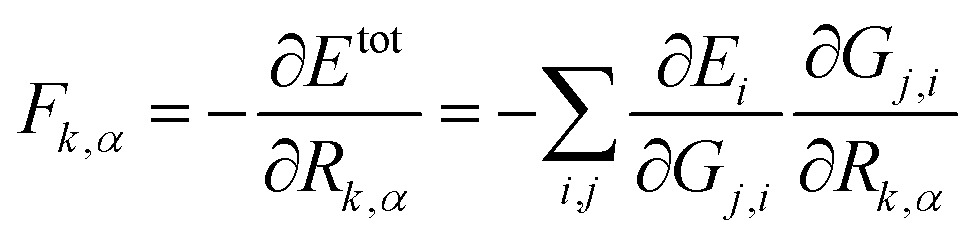



For solid systems, an accurate static stress tensor is critical in structure optimization and thus for converging total energy. Similarly, the static stress tensor matrix element *σ*_*αβ*_ can be analytically derived as:9
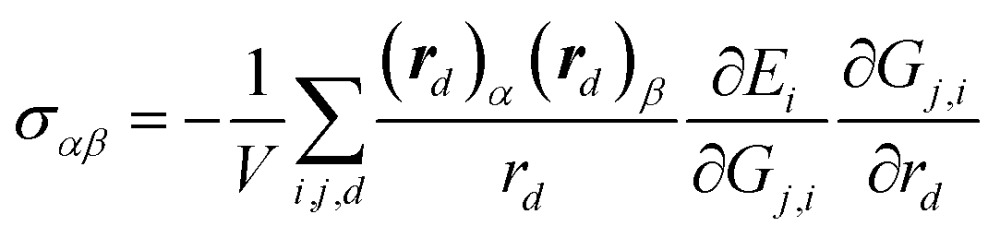
where ***r***_*d*_ and *r*_*d*_ are the distance vector constituting of *G*_*j*,*i*_ and its module, respectively, and *V* is the volume of the structure.

#### Neural network training

Once the network architecture is settled, the next step is to determine the weights and biases (NN parameters) in each NN subnet, a process known as the training of the NN. The number of weights and biases is typically 10^4^ to 10^6^ for the standard feedforward NN with two hidden layers, each with 40 nodes (for most systems ∼200 ACSFs are used to depict a single atom). In order to train such a large number of NN parameters, a performance function *J*_tot_ first needs to be defined, which measures the deviation of NN output with respect to the training set properties. The training process will minimize *J*_tot_ until the accuracy of the NN predicted properties reaches the preset criteria.10
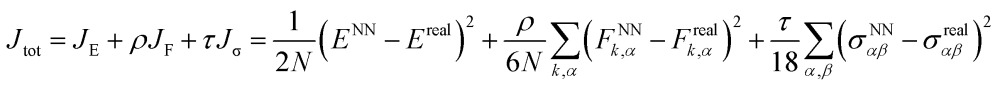



One major feature in our NN training is the incorporation of the stress tensor into the performance function (eqn (10)), which allows us to fit all three properties simultaneously. Pukrittayakamee *et al.* ^21^ first introduced the mixed scheme to simultaneously fit energy and force, and found that the mixed scheme can avoid overfitting, produce more accurate forces and thus improve the predictive ability. In our practice, we found that for the global optimization of solids, both forces and stresses need to be accurate and the most convenient way is to allow the NN training to fit all three terms, either simultaneously or independently (depending on the adjustable parameters, *ρ* and *τ*, in eqn (10)).

To train the NN, the derivatives of each term in eqn (10) with respect to the weights and biases need to be analytically derived. Among them, the gradient of *J*_E_ can be computed by the standard backpropagation algorithm,^27^ and the gradient for *J*_F_ has been reported in ref. 21. We have derived equations to compute the gradient of *J*_σ_, which are detailed in the ESI.†

In this work, we utilize the limited-memory Broyden–Fletcher–Goldfarb–Shanno (L-BFGS)^28,29^ method to minimize *J*_tot_, which is convenient for massive parallel programming. The performance of the L-BFGS method has been demonstrated in fitting complex NNs.^30^ For the training set of ∼50 000 structures from our SSW global optimization, we found that L-BFGS takes typically 20 000 training epochs to generate a qualified PES with the root mean square errors (RMSEs) for energy, force, and stress being around 10 meV per atom, 150 meV Å^–1^, and 1.5 GPa, respectively.

We note that many other gradient-based optimization algorithms have been used to optimize the network weights and biases, such as stochastic gradient descent (SGD),^31^ conjugate gradient (CG),^32,33^ Levenberg–Marquardt (LM)^20^ *etc.* It is in general accepted that quasi-Newton second-order methods, such as L-BFGS and LM, converge more rapidly to the true minimum for large data sets. It should be mentioned that compared to the dominant computational effort to generate the data set using quantum mechanics calculations, the training of the NN is in fact not the rate determining step in the whole NN PES construction procedure.

### b. Automated data set generation from stochastic surface walking global optimization

Undoubtedly, the data set used for training the NN largely determines the quality of the NN PES. Previous work utilizes either local PES data, *e.g.* the MD trajectories of a single reaction,^16,34^ or the combination of local PES data from different structure sources, *e.g.* solids, slabs and clusters.^14^ This work aims to increase the transferability of the NN PES as well as to simplify the data set generation procedure. In this regard, the structures produced from global optimization trajectories appear to be the natural choice, which should be able to represent as differently as possible the chemical environment of atoms.

To date, there are a variety of global optimization methods for structure prediction, including, for example, simulated annealing,^35^ basin-hopping,^2–4^ minimum-hopping,^36^ genetic algorithm^7–10^ and the SSW method,^11,12^ which should be able to search a wide area on the PES, and identify unbiasedly the global minimum (GM) even starting from random structures. Among them, basin-hopping and genetic algorithms transform the PES by overlooking the transition region between minima and thus they are well likely to miss important structural patterns at the transition region. On the other hand, simulated annealing and minimum-hopping are based on extensive MD calculations at elevated temperatures, and both methods are rarely utilized for global search in combination with quantum mechanics (even DFT) calculations. Furthermore, the structure patterns from the MD trajectories being closely related could be overwhelmingly redundant for NN training and an iterative structure selection scheme may be required to produce a compact data set.^13,17^

In this work we decided to utilize the SSW method developed in the group to generate the global PES structure data set. The SSW method in combination with DFT calculations has been utilized in a number of complex systems, ranging from clusters^37–39^ to surfaces^40^ and to solids.^41^ This allows us to sample different PESs within the same theoretical framework. The methodology of SSW has been detailed in our previous work,^11,12,37^ and here we provide a brief summary for the working mechanism of SSW (the algorithm of SSW is also described in the ESI†).

The SSW method targets for a rapid PES exploration *via* smooth structure perturbation. The idea of the SSW method comes from the bias-potential driven dynamics^42^ and Metropolis Monte Carlo sampling. Specifically, SSW smoothly manipulates the structural configuration on the PES from one minimum to another by adding bias potentials along a softened random direction and relies on the Metropolis Monte Carlo to accept or refuse each move. Thus, the SSW trajectories (see ESI Fig. S1†) include a variety of structural configurations on the PES ranging from minimum to saddle points and even to fragmented/open structures with high energy. Due to the continually-added bias potentials, even high barriers separating the minima on the PES can be surmounted efficiently. This exploration scheme is fully automated and does not need *a priori* knowledge of the system, such as the structure motif (*e.g.*, bonding patterns, symmetry) of materials.

**Scheme 1:**
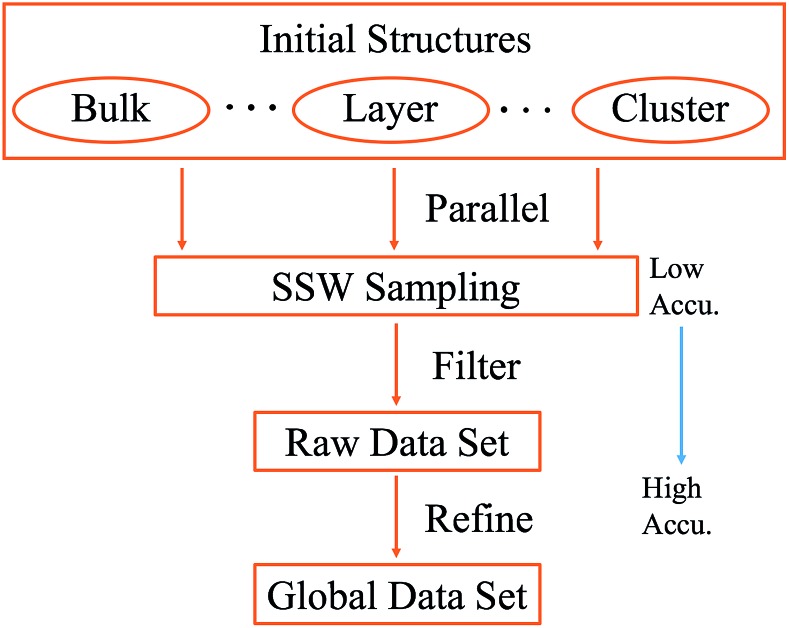
Procedure for generating the training data set using SSW global optimization. The SSW sampling is typically calculated with low accuracy quantum mechanics calculations, while the final global data set is further refined using high accuracy setups.

The SSW method for PES exploration is massively parallel in nature: in practice we performed a series of SSW simulations starting from different initial structures using DFT calculations. To generate a representative training set for a global PES, it is ideal to initialize SSW simulations from different phases, including the solid and layer phases and clusters. Nevertheless, this is not obliged, especially for unknown systems, since SSW being a global optimization method can visit stochastically a broad area on the PES as the SSW steps increase. For the purpose of material discovery concerned here, we will focus on solid and layer types of structures, which are treated in the same framework of periodic plane-wave DFT. In Scheme 1, we summarize the procedure for generating the data set using SSW and it is explained as follows.

(i) Set the initial structure for SSW simulations; a group of initial structures is randomly selected to represent solids, layers and clusters.

(ii) Sample the global PES using SSW in parallel; typically, DFT calculations with low accuracy setups are utilized to speed up the PES sampling. All structures along the SSW trajectories are recorded, which yields the raw data set.

(iii) Filter and generate the reference data set; the raw data set needs to be screened according to energy and geometry to remove redundant and too-high energy structures. For the large number of structures in the raw data set (typically more than 200 000 structures), a smaller data set is randomly selected from the filtered raw data set, which yields the reference data set.

(iv) Refine energy, forces and stresses for the reference data set. The structures in the reference data set will be recalculated (single point energy) using DFT (or high level quantum mechanics) calculations with high accuracy setups.

### c. Example of global PES

To illustrate a global DFT PES mapped out by SSW simulations, we take a TiO_2_ system as an example. TiO_2_ as an important functional material is widely utilized as coating materials and catalysts in many fields. In particular, the photoactivity of TiO_2_ was hotly investigated for water splitting.^43–46^ The PES of TiO_2_ is highly complex as evident by the fact that many possible polymorphs are present in nature (the most common phases of TiO_2_ at the ambient conditions are rutile, anatase, brookite, and TiO_2_-B^47–49^). New stable TiO_2_ phases have been pursued for years in material science.

In order to generate the DFT global PES, we run ten SSW simulations in total, 8 of them starting from bulk solids and 2 of them starting from layer structures where one dimension of the lattice is fixed to maintain a large vacuum (>10 Å) between adjacent unit cells. The system size is selected as 12 atoms (Ti_4_O_8_) and the total SSW steps for each run are limited to within 100 steps, which is suitable for DFT calculations and at the same time for enabling the SSW sampling to produce enough different structural patterns (including amorphous structural patterns). The starting TiO_2_ structures of SSW are either known crystal structures (*e.g.* rutile), or randomly configured. All DFT calculations performed in this work are based on the periodic plane-wave framework, as implemented in VASP,^50,51^ and the DFT setups are detailed in the ESI.†

**Fig. 2:**
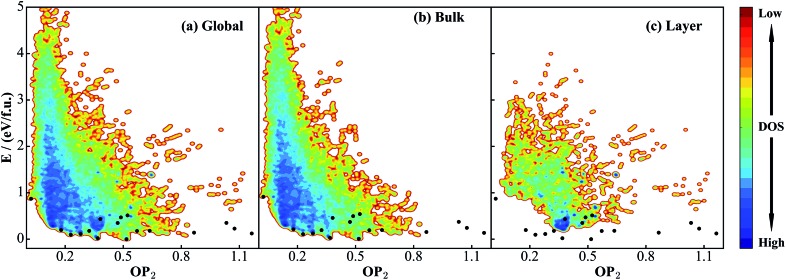
Illustration of global DFT PES for TiO_2_ solids using the OP_2_ ∼ *E* contour plot. OP_2_: the structural order parameter *l* = 2 (eqn (11)). The energy of the GM (TiO_2_-B phase) is set as energy zero. (a) Global data set constructed according to the procedure described in Scheme 1. (b) and (c) are the data sets taken from SSW simulations starting from bulk initial structures and layer initial structures, respectively. Both are part of the global data set in (a). The black dots label the minima listed in Table 1 that are identified from the SSW global structural search using global NN PES and reoptimized using DFT. Their coordinates (*E*, OP_2_) in the figures are as follow: TiO_2_-B: (0, 0.52); anatase: (0.02, 0.37); TiO_2_-II: (0.09, 0.23); rutile: (0.11, 0.28); Str-1: (0.12, 1.16); Str-2: (0.14, 0.87); TiO_2_-R: (0.18, 0.32); TiO_2_-H: (0.18, 0.64); lepidocrocite: (0.18, 0.57); baddeleyite: (0.20, 0.18); Str-3: (0.23, 1.07); Str-4: (0.35, 0.47); Str-5: (0.35, 1.03); Str-6: (0.43, 0.38); Str-7: (0.47, 0.49); Str-8: (0.51, 0.52); TiO_2_-OII: (0.87, 0.02).

Fig. 2a shows the global PES constructed using the 50 000 structures, which are filtered and randomly selected from a 130 000-structure raw data set. To project the global PES into two dimensions for visualization, *i.e.* energy *versus* geometry, here we distinguish the geometry of structure using the distance-weighted Steinhardt-type order parameter (OP),^52^ presented on the *x*-axis of Fig. 2. The *y*-axis is the energy per formula unit (f.u.), where the energy zero is set as that of GM (TiO_2_-B phase) hereafter. The OP is written as:11
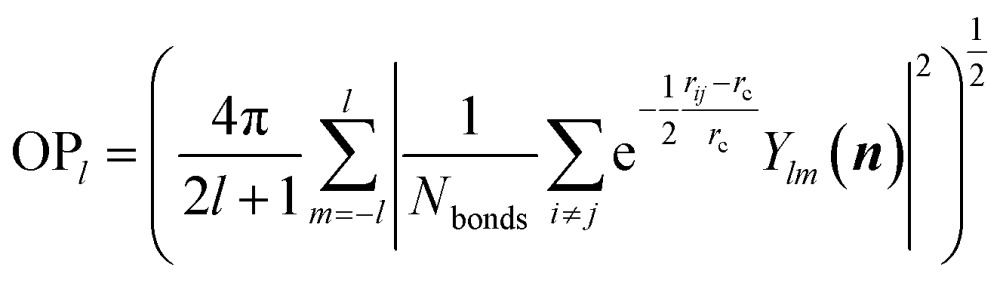
where *i* and *j* are the atoms in lattice; *r*_*ij*_ is the distance between atoms *i* and *j*; *r*_c_ is set as 60% of the typical single bond length for the *i* and *j* atoms; *N*_bonds_ is the number of bonds in the first bonding shell and the degree *l* is set as 2 (OP_2_).

As seen from Fig. 2a, the stable crystal phases are located at the bottom of the global PES. The OP_2_ values for the four common crystal phases, TiO_2_-B, anatase, rutile, and TiO_2_-II as highlighted by the black dots, are 0.52, 0.37, 0.28, and 0.23, respectively. It is obvious that most of the structures from the SSW trajectories are located nearby the anatase and rutile phases with the OP_2_ value being 0.2–0.4. This may not be surprising since these two phases contain only TiO_6_ octahedra that are the most common structural patterns in TiO_2_ crystals.

Since this DFT global PES is glued together from all SSW trajectories, it is of interest to examine the contributions from different initial structure sources. Fig. 2b and c show the bulk-starting PES and layer-starting PES, respectively. Interestingly, the former covers most parts of the PES and is quite similar to the global PES, while the latter concentrates mainly at the middle region of the OP_2_-energy plot. It should be noted that due to the limited SSW sampling steps (100 SSW steps), the bulk-starting SSW trajectories have a low probability of visiting some of the layer structures, as evident by the lack of population in the region of large OP_2_ (>0.9). On the other hand, the layer-starting SSW simulation is constrained to keep the vacuum in the *Z*-direction and thus the common bulk crystal phases are not found in the trajectories (*e.g.* see Fig. 2c for the black dots).

## 3. Results and discussions

With the above DFT global PES, it is now possible to construct the NN PES of TiO_2_ by NN training. To this end, the DFT global data set shown in Fig. 2a is first split into two parts: 90% of it is taken as a training set and the remaining 10% as a test set. We utilize the standard NN with two hidden layers for each element-based subnet, and each layer contains 40 nodes. The input nodes are based on ACSFs (eqn (2)–(7)) and all of the parameters for Ti- and O-ACSF are listed in the ESI.† Specifically, two different cutoff radii (*R*_c_ in eqn (2)), 3.3 and 6 Å, are utilized in generating the ACSFs, which is found to be beneficial to the training of forces and stresses (the comparison for different numbers of cutoff radii is shown in ESI Fig. S2†). In total, the O element subnet has 86 symmetry functions and the Ti element subnet has 80 functions (thus the NN architecture for the O and Ti elements is 86-40-40-1 and 80-40-40-1 in the standard notation, respectively).

It should be mentioned that ACSFs depict only the short/medium-range atomic environment (defined by the cutoff radius) and thus the long-range electrostatic interaction, if present in bulk materials, *e.g.* ionic solids, may dominate the error of the NN. Considering the strong ionic nature of TiO_2_, we have decided to utilize both the fixed charge model by setting the O and Ti atom as –0.5|*e*| and 1.0|*e*|, respectively, and the floating charge model as introduced by Behler.^14^ These atomic charges will be utilized for solving the Ewald electrostatic energy^53^ of a crystal to account for the long-range electrostatic interactions and the total energy for NN training (eqn (1)) is modified as

12 *E*^tot^ = *E*^DFT^ – *E*^Ewald^

which is the difference between the DFT total energy and the Ewald energy. We found that the exact atomic charge (*e.g.* O charge ranging from –0.3 to –0.7) appears to be not critical for the overall quality of the NN PES considering that the fixed charge model can already produce accurate energies for the global optimization of TiO_2_ bulk material (as demonstrated below). This could be due to the fact that the error introduced by the fixed charge model can be cancelled largely in the NN training.

### a. NN training and performance of NN PES

Our NN training is fully parallel with the data set distributed evenly into hundreds of CPU cores. By minimizing the performance function, the L-BFGS method enables the energy, forces and stresses from the NN to approach simultaneously to those from the DFT calculations. The training is terminated when the RMSEs for energy, force and stress for the training set reach 28 meV f.u.^–1^ (9.6 meV per atom), 132.2 meV Å^–1^, and 1.10 GPa, respectively (test set: 27 meV f.u.^–1^ (9.0 meV per atom), 125.9 meV Å^–1^ and 1.02 GPa). Considering that the data set spans an energy window of 7.2 eV f.u.^–1^, the accuracy of the current NN PES is satisfactory and enough for global optimization.

**Fig. 3:**
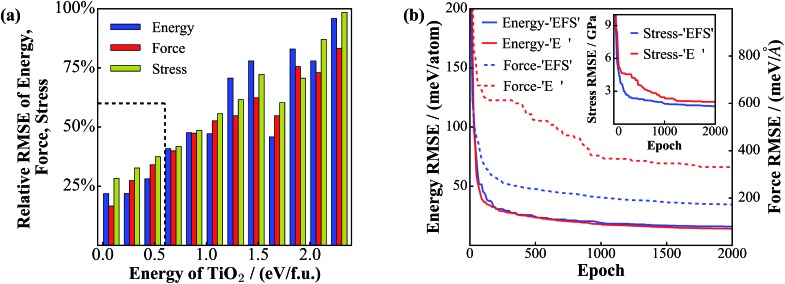
Performance of the NN global PES and the convergence of NN training. (a) Energy-resolved RMSEs of energy, force and stress for the test set. The *x*-axis is the energy (eV f.u.^–1^) of the TiO_2_ phases with the energy zero being that of the GM (TiO_2_-B phase). The maximum values (100%) of the *y*-axis correspond to an energy value of 48 (meV f.u.^–1^), a force value of 240 meV Å^–1^ and a stress value of 1.6 GPa. The energy below ∼0.6 eV f.u.^–1^ as rectangulated by the dotted lines is the most important region, including crystals and low energy amorphous structures. (b) Evolution of energy and force RMSEs for the training set during the NN training. ‘*EFS*’ and ‘*E*’ stand for the simultaneous energy/force/stress training and the energy-only training, respectively. The inset shows the convergence of the stress RMSE.

Fig. 3a shows the energy-resolved NN performance, including the RMSEs of energy, force, and stress for the test set, where the RMSEs of the structures in the same energy interval, *E* to *E* + d*E* (0.20 eV f.u.^–1^), are calculated. It is clear that the RMSEs increase as the energy increases, which suggests simply that the low energy structures can be predicted much more accurately compared to the high energy structures. This might be understood as follows. First, the predictive ability of the NN is much dependent on the density of the data set: the low energy structures are more frequently sampled due to the Metropolis Monte Carlo algorithm. Second, compared to the low energy structures, the high energy structures, often related to defected crystals, saddle states and liquid-like structures, have a rich structural variety and a high density of states. The sampling of these high energy structures is technically difficult and insufficient, even with the SSW global optimization method.

Nevertheless, for the most important energy region of materials, ∼0.6 eV f.u.^–1^ (*e.g.* the rutile-anatase solid phase transition barrier of TiO_2_ is calculated to be ∼0.3 eV f.u.^–1^ that occurs above 500 K in experiment^40,54^), the NN performance is rather accurate with low deviations, which are ∼12 meV f.u.^–1^ (4 meV per atom), 60 meV Å^–1^ and 0.4 GPa for the RMSE of energy, force and stress, respectively. This provides us with great confidence for both new structure discovery and reaction pathway sampling (shown in the next section).

To illustrate the fast convergence for the L-BFGS simultaneous fitting of energy, force and stress (*EFS* training), we have compared the convergence curve with that from fitting the energy only (*E* training) as is done traditionally. This is shown in Fig. 3b, where the blue and red lines represent the *EFS* and *E* training, respectively (solid and dotted lines for the minimization of energy and force, respectively, as measured by the RMSE values). While both training sets show similar convergence for the energy RMSE, the performance for the force and stress (shown in the inset) is obviously better in the *EFS* training. Especially, the force RMSE in the *EFS* training after 2000 epochs is 173 meV Å^–1^, which is about half of that in the *E* training (334 meV Å^–1^). We emphasize that the forces and stresses are critical for global optimization: (i) they are required in the structural optimization to reach to the true local minima and also the saddle points; (ii) they determine the structural perturbation direction in the SSW global optimization and thus control the efficiency for PES exploration. The SSW method utilizes a numerical method, the constrained Broyden dimer method,^55^ to soften the randomly generated mode, along which the structure configuration is pushed out of the local minimum.

Finally, it might be mentioned that the obtained NN PES calculations are computationally much less demanding than DFT calculations. The NN PES calculations scale linearly with the increase of the system size due to the parallel nature of the HDNN scheme (partitioning the total energy into the contribution from individual atoms). We have compared the performance of the NN PES calculations for different sized TiO_2_ systems with that using DFT calculations. For TiO_2_ solids of medium size (48 atoms), the NN PES calculation is ∼20 000 times faster than the DFT calculations (ESI Fig. S2 and Table S7†). With the high efficiency of the NN PES, it is now feasible to explore the global PES of large systems using the SSW method, which is practically forbidden in the framework of quantum mechanics calculations (see the example below, and the comparison of the computational cost is detailed in ESI†).

### b. Influence of data set on NN PES

The quality of the training set will largely determine the predictive power of the NN PES. Ideally, the training set needs to incorporate structural patterns that are as varied as possible, *e.g.* different polyhedron coordination and different linking motifs (cage, ring and lines). It is of significance to ask whether the current DFT global data set (Fig. 2a) is representative enough for TiO_2_ solids. To answer this, we have taken two steps to verify the predictive ability of the NN PES.

**Fig. 4:**
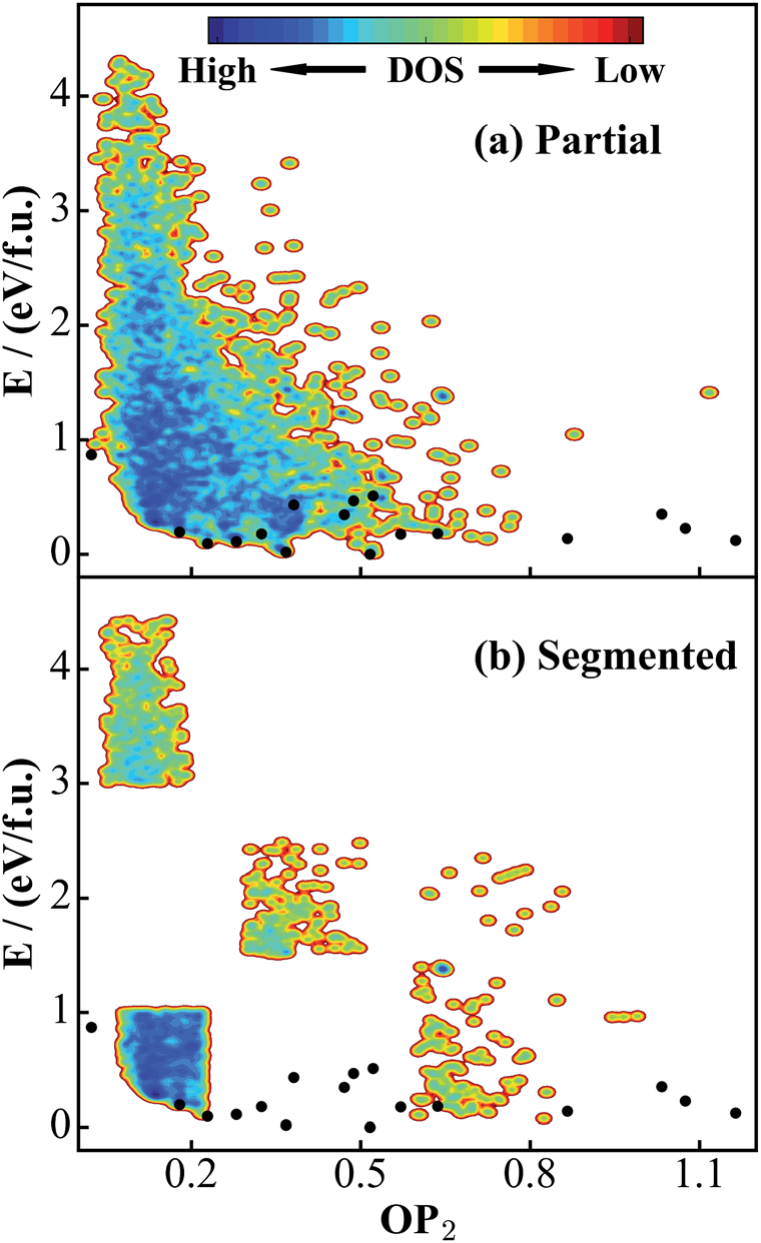
PES contour plots for two small DFT data sets truncated from the global data set in Fig. 2a. (a) Partial data set: 4000 structures randomly selected from the global set. (b) Segmented data set: 4000 structures taken randomly from selected OP_2_-energy regions in the global set. Also see the Fig. 2 caption for the meaning of the black dots.

First, based on the global set we construct another two smaller data sets, as shown in Fig. 4: (a) a partial set, which contains only 4000 structures that are randomly selected from the global set; (b) a segmented set, which also contains only 4000 structures taken randomly from selected OP_2_-energy regions (see Fig. 4b). The segmented set is thus highly discontinuous. We then follow the same NN training procedure to train these two data sets and obtain the corresponding NN PES, denoted as the partial NN PES and segmented NN PES.

Second, we run the SSW global optimization with a 12-atom unit cell using the global NN PES. All experimentally known structures (9 structures, their structural parameters are listed in the ESI†) in the 12-atom unit cell have been found from SSW. In Table 1, we compare the performance of the global, partial and segmented NN PES for these known structures and other 8 randomly selected low energy structures within the 0–0.6 eV f.u.^–1^ window (Str-1 to Str-8), which are benchmarked against the DFT calculations results. All of these structures have been locally optimized using different PESs until the maximal force component is below 0.01 eV Å^–1^ and the stress is below 0.01 GPa (the same criteria for minimum convergence are utilized throughout the work). It is noted that Str-1, 2, 3, and 5 are newly found structures from the 12-atom SSW-NN that are not present in the global DFT PES in Fig. 2. This is obviously due to the fact that the SSW sampling in DFT only runs limited steps.

**Table 1:** Comparison of the energy and volume of selected low energy structures of TiO_2_ calculated using different NN PES and DFT calculations. All energies are with reference to that of GM (TiO_2_-B)

No	Species	SG^*a*^	OP_2_ ^*a*^	*E*_DFT_/eV f.u.^–1^	d*E*/(eV f.u.^–1^)^*b*^			*V*_DFT_/Å^3^ f.u.^–1^	d*V* ^*b*^/%		
					Global^*d*^	Part.	Seg.		Global	Part.	Seg.
1	TiO_2_-B	12	0.52	0.00	–0.01	0.01	–0.01	36.66	0.43	0.38	0.34
2	Anatase	141	0.37	0.02	–0.01	0.00	0.03	35.19	–0.03	0.57	0.93
3	TiO_2_-II	60	0.23	0.09	0.01	0.02	0.04	31.54	0.59	0.01	0.21
4	Rutile	136	0.28	0.11	0.00	–0.00	0.03	32.15	0.14	–0.32	–0.26
5	Str-1	74	1.16	0.12	0.01	0.08	0.17	83.16	–0.39	–0.82	1.25
6	Str-2	2	0.87	0.14	0.01	0.03	–0.06	52.88	2.26	1.75	–8.13
7	TiO_2_-R	62	0.32	0.18	0.01	0.02	0.00	35.31	0.94	–0.32	3.05
8	TiO_2_-H	87	0.64	0.18	0.04	0.02	–0.02	39.56	0.80	0.10	0.29
9	Lepidocrocite^57^	59	0.57	0.18	–0.00	0.01	0.01	44.75	0.12	–0.68	–0.12
10	Baddeleyite	14	0.18	0.20	0.02	0.03	0.02	29.89	0.03	–0.01	0.07
11	Str-3	5	1.07	0.23	–0.02	0.09	0.12	76.19	0.49	4.22	3.43
12	Str-4	12	0.47	0.35	0.05	–0.05	–0.09	37.62	0.99	17.11	16.93
13	Str-5	5	1.03	0.35	0.00	0.11	0.17	78.33	1.07	2.25	2.81
14	Str-6	12	0.38	0.43	0.03	–0.13	–0.14	33.35	0.82	18.37	15.67
15	Str-7	6	0.49	0.47	0.01	–0.09	–0.13	39.91	–1.11	6.97	6.21
16	Str-8	1	0.52	0.51	–0.00	–0.02	–0.05	41.04	3.18	4.07	8.74
17	TiO_2_-OII	62	0.02	0.87	–0.03	–0.80	–0.82	26.25	0.49	96.88	35.36
	RMSE				0.02	0.20	0.21		0.94	22.72	9.55
					(0.02)^*c*^	(0.06)^*c*^	(0.09)^*c*^		(0.97)^*c*^	(5.81)^*c*^	(6.02)^*c*^

^*a*^SG: symmetry group; OP_2_: Steinhardt-type order parameter (see eqn (11)).

^*b*^d*E* and d*V*: deviation of energy and volume in percentage from the NN PES with respect to those (*E*_DFT_ and *V*_DFT_) from the DFT PES, respectively.

^*c*^The data in brackets are the RMSE excluding the data of the TiO_2_-OII phase.

^*d*^Global, part. and seg. refer to the NN PES trained from the global DFT PES (Fig. 2a), the partial DFT PES (Fig. 4a) and the segmented DFT PES (Fig. 4b), respectively.

In Table 1, we show the energy and volume for these energy minima computed from different PESs. As shown, the overall performance of the global NN PES is the best with an energy RMSE of 0.02 eV f.u.^–1^ and a volume RMSE of 0.94%, while the segmented NN PES provides the worst results with the energy and volume RMSE being 0.21 eV f.u.^–1^ and 9.55% (including the TiO_2_-OII phase). The energy sequence for the lowest six minima from DFT is TiO_2_-B < anatase < TiO_2_-II < rutile < Str-1 < Str-2, which is correctly predicted only by the global NN PES. The segmented NN PES, for example, wrongly predicts TiO_2_-B < anatase < Str-2 < TiO_2_-II < rutile < Str-1.

It is noted that three structures, Str-1, 3, and 5, are poorly predicted by partial and segmented NN PESs. In particular, the high pressure phase TiO_2_-OII is not stable on these two PESs. All Ti atoms in Str-1, 3, and 5 are in a 4-coordinated polyhedron [TiO_4_], while that in TiO_2_-OII is [TiO_8_], which is a rare coordination environment in TiO_2_ solids (the percentage of structures containing 4/8-coordinated Ti in all data sets is about 2% and 5%, as compared to 45% for the common 6-coordinated Ti). Because the size of the global set is approximately 10 times larger than that of the other two data sets, only the global set contains a sufficiently large number of structures containing [TiO_4_] and [TiO_8_]. We expect that this helps to greatly improve the predictive ability in the global NN PES. From the comparison in Table 1, we conclude that both the size and the structural varieties of the DFT data set will affect the predictive ability of the NN PES. This reaffirms the necessity of applying SSW global optimization for global data set generation.

## 4. Application of NN PES for material discovery

### a. New TiO_2_ solid phases from global PES

**Fig. 5:**
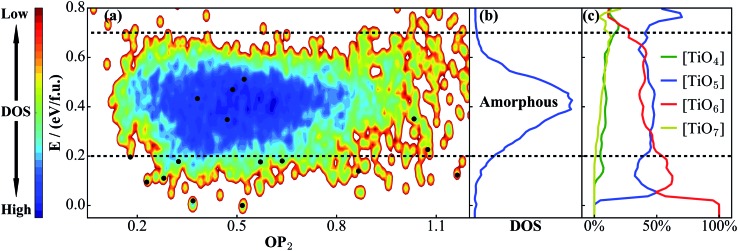
(a) PES contour plot for TiO_2_ distinct minima sampled from the SSW-NN global search. (b) Density of states (DOS) plot for TiO_2_ phases in (a), showing that amorphous structures appear in the 0.2–0.7 eV f.u.^–1^ window. (c) The percentage of differently coordinated Ti for TiO_2_ structures. [TiO_5_] and [TiO_6_] are the main structural features for amorphous structures. Also see the Fig. 2 caption for the meaning of the black dots.

We are now in a position to exploit the TiO_2_ NN PES for material discovery. A natural application is to combine SSW with NN PES to explore the global PES of large TiO_2_ systems, from which new TiO_2_ phases with desirable properties can be identified. To test this idea, we have carried out six independent SSW global search for 48-atom supercell TiO_2_ systems, with 10 000 minima to visit for each run (in fact, even for a 48-atom supercell TiO_2_ system, it is practically infeasible using DFT to properly sample the PES since the complexity of the PES increases exponentially as the atom number grows). After removing the duplicated ones, we finally obtain 6151 distinct minima. The OP_2_-energy PES contour plot for these minima is shown in Fig. 5a and the corresponding density of states (DOS) are plotted in Fig. 5b.

The PES in Fig. 5a shows that the crystal minima are rather scattered below ∼0.2 eV f.u.^–1^, which in total contains 224 minima in the 48-atom system (among these minima, 180 minima are newly found from the SSW-NN simulation). These belong generally to single- and mixed-phase crystalline structures. We note that the number of crystalline minima is substantially large considering that there are only ∼10 TiO_2_ phases reported from experiment.^49^ From 0.2 to 0.7 eV f.u.^–1^, one can see a salient deep-blue zone due to the energy degeneracy of the structures, featuring a high, continuous and broad peak in the DOS plot (Fig. 5b). This zone is dominated by amorphous structures.

It is also of interest to analyze the geometrical structure for all of these TiO_2_ minima, which could help to clarify the difference between amorphous and crystalline structures. For this purpose, we have computed the Ti coordination number *x* ([TiO_*x*_]) for all structures. In Fig. 5c, we plot the evolution of the Ti coordination environment with the increase of energy. This is done by counting and averaging the Ti coordination for structures in the same energy interval, *E* to *E* + d*E* (5 meV f.u.^–1^). As shown, [TiO_4_], [TiO_5_], [TiO_6_] and [TiO_7_] are the major coordination environments for Ti. For the low energy crystal phases (*e.g.* rutile, anatase, and TiO_2_-B), they generally have only TiO_6_ octahedra (100% [TiO_6_], energy lower than 25 meV f.u.^–1^). The [TiO_5_] starts to appear above 30 meV f.u.^–1^ and it together with [TiO_6_] is the dominant coordination for amorphous structures. By contrast, [TiO_4_] and [TiO_7_] are rare coordinations in TiO_2_ solids: they appear at high energies and are found either in high pressure phases ([TiO_7_]) or in open (*e.g.* cage, 2-D layer) structures ([TiO_4_]) along with low coordination O atoms.

**Fig. 6:**
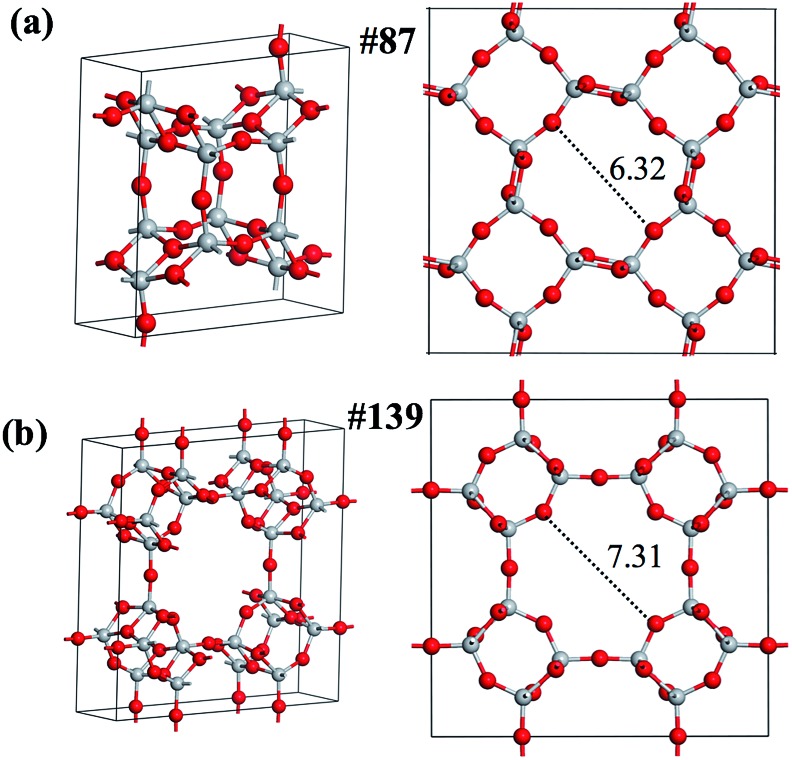
Two new porous TiO_2_ phases identified from the SSW-NN global search. (a) Phase-87; (b) phase-139. The Bravais lattices are shown on the left and the pore geometries with the pore size labeled are shown on the right.

**Fig. 7:**
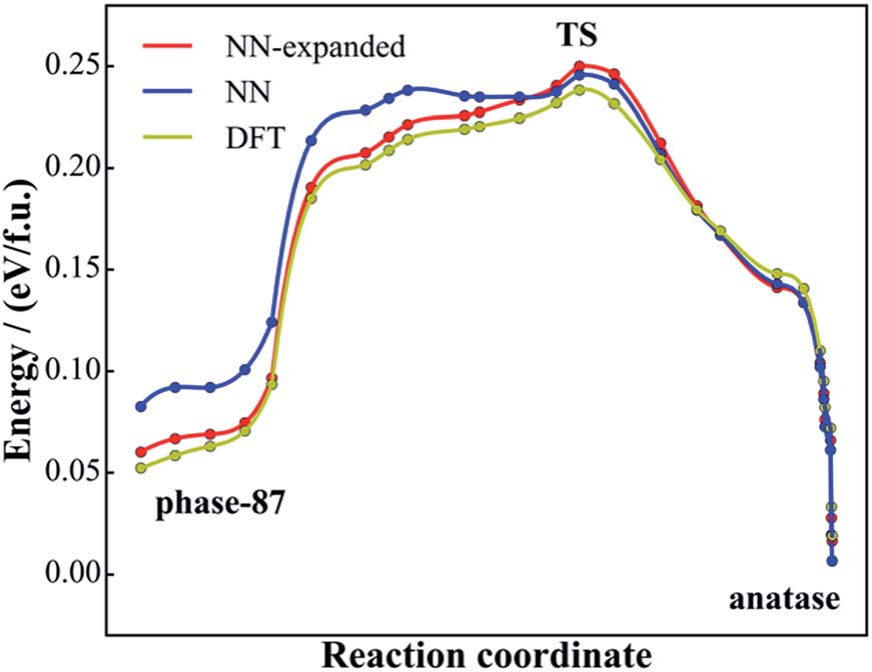
Minimum energy reaction pathway for the solid-to-solid phase transition from phase-87 to anatase using DFT and NN PES. The reaction coordinate is the relative distance in the generalized coordinate containing both the lattice and atom (see ref. 56 for detail) with respect to the initial state and the final state.

We then focus on the low energy crystalline structures to search for stable porous structures, which could be potentially useful as molecular sieves and catalysts. Indeed, we find two such phases, labeled as phase-87 (*I*4/*m*, #87) and phase-139 (*I*4/*mmm*, #139), which rank 10 and 23 in the minimum spectrum, respectively, as shown in Fig. 6. These two phases are alike and have open cages (their structures are detailed in the ESI†), are not known previously and are also not identified in Fig. 2 (12-atom PES using SSW-DFT). As shown in Table 2, both phases are unexpectedly stable with the calculated total energy comparable to the common TiO_2_ rutile phase (0.11 eV f.u.^–1^), which ranks 30 in the minimum spectrum. It might be mentioned that the NN PES has correctly predicted the energy and geometry of these phases: the energy, pore size (labeled in Fig. 6) and volume deviations are smaller than 0.02 eV f.u.^–1^, 0.08 Å and 0.7 Å^3^ f.u.^–1^, respectively, compared to the DFT results, as detailed in Table 2. Because of their high thermodynamic (as reflected from the energetics) and kinetics stability (as seen from the pathway in Fig. 7), further studies are carried out in the group to explore the synthetic possibility of these porous phases.

**Table 2:** Comparison of energy (eV f.u.^–1^), pore size (Å), and volume (Å^3^ f.u.^–1^) of two TiO_2_ phases between the DFT and global NN PES calculations, as shown in Fig. 6

Phase	*Z*	Energy		Pore size		Volume	
		DFT	NN	DFT	NN	DFT	NN
87	8	0.05	0.07	6.32	6.40	53.86	54.21
139	16	0.10	0.10	7.31	7.35	57.68	57.90

### b. Pathway sampling to reveal solid-to-solid phase transition pathways

Compared to the PES from traditional force fields, the NN PES has an attractive perspective for describing chemical reactions, where chemical bonds are made and broken. For solids, this would provide a new opportunity to quickly predict the kinetics stability of a solid phase if the solid-to-solid phase transition pathways can be determined with low computational costs. We have shown previously^41^ that SSW reaction pathway sampling is able to sample the solid phase pathways and, by combining with the variable-cell double-ended transition state (TS) search method,^56^ it is likely to identify the lowest energy pathways for solid-to-solid phase transition. Because the DFT based SSW pathway sampling is highly computationally demanding, this approach was still limited for systems with less than 24 atoms.^41^ Obviously, the application of NN PES in this field can significantly expedite the research on solid-to-solid phase transitions.

Taking the newly found porous phase, phase-87 (Fig. 6a), as the example, we have examined its kinetics stability by searching for the lowest energy pathways linking it with other phases. SSW pathway sampling was performed similarly as described in the previous work.^52^ In short, the SSW starts from phase-87 and explores the likely product phases nearby. All of the product phases that are different from phase-87 will be recorded, which yields a database of reactant/product pairs. In total, 170 reactant/product pairs were obtained after SSW visits to 1000 minima. By using the variable-cell double-ended surface walking (VC-DESW) method,^56^ we then searched the TS of all pathways and found that the lowest energy pathway escaping from phase-87 leads to the anatase phase.

Fig. 7 shows the reaction profiles for the lowest energy pathway from phase-87 to anatase using both NN PES (blue line) and DFT (yellow). As shown, the reaction profile from NN PES agrees generally well with that from DFT, where the maximum deviation in energy is less than 34 meV f.u.^–1^ and the energies of the TSs in the two profiles are nearly the same (∼8 meV f.u.^–1^). The accuracy at the phase-87 side (30 meV f.u.^–1^) is poorer than that at the anatase side (12 meV f.u.^–1^), apparently because phase-87 as a new phase is not present in the DFT training set.

Importantly, the calculated barrier for the phase-87 to anatase transition is 0.22 eV f.u.^–1^, which is quite high considering that the barrier from TiO_2_-B to anatase is only 0.12 eV f.u.^–1^ and the experimental temperature for TiO_2_-B to anatase conversion is ∼673 K.^54^ Our results indicate that the porous phase-87 is kinetically stable. It is well likely to stabilize the new phase at ambient conditions once it can be synthesized.

Finally, it might be mentioned that the NN PES can be improved once new structure data with quantum mechanics calculations are added into the training set. In this work, after SSW pathway sampling on phase-87, we add 2000 new structures from the sampling trajectories (calculated using DFT with high accuracy setups) into the global data set, and follow the same training procedure to train the NN PES. Using the re-trained NN PES, we recalculate the reaction pathway, as shown by the red line in Fig. 7, and find that the pathway using the expanded NN PES matches with the DFT pathway nicely, with the maximal energy deviation smaller than 12 meV f.u.^–1^ These results indicate that NN PES generated from DFT global PES, although it has a good predictive ability for both structure search and pathway sampling, can be further improved once new structures or pathways are identified. For the purpose of material discovery, such an improvement, however, might not be essential since the quantum mechanics calculations can always be performed afterwards for validation.

## 5. Conclusion

This work develops a general-purpose theoretical framework for exploring the complex PES of materials, which combines the SSW global optimization method with the high dimensional NN. The SSW-NN method constructs a global NN PES for a material, based on the global data set generated from SSW PES sampling. As this procedure is automated and massively parallel, it largely reduces the efforts required traditionally in iteratively training NNs *via* gradually expanding local data sets. The global NN PES is robust, transferable and ready for use both in the global structure search and the reaction pathway sampling of large systems. The highlights of the current SSW-NN implementation are as follows.

(i) The data set is generated using a SSW global structural search that runs in parallel from varied initial structures. These SSW simulations are carried out typically in relatively small systems under a periodic DFT framework.

(ii) The NN training of the data set utilizes a performance function constituting energy, forces and stresses, which is minimized using the L-BFGS method to achieve a fast convergence.

(iii) The global NN PES matches the DFT PES in a wide energy window (∼7 eV f.u.^–1^) with regard to total energy, forces and stresses. The accuracy of the NN PES drops slowly with the increase of energy. For a medium size system (*e.g.* 48 atoms), the global NN PES is ∼four orders of magnitude faster than DFT.

Using the SSW-NN method, the global NN PES for TiO_2_ solids is constructed. In the most concerned energy region (*e.g.* ∼0.6 eV f.u.^–1^ above GM), the typical accuracy in RMSE is within 12 meV f.u.^–1^ (4 meV per atom) for energy, 60 meV Å^–1^ for force and 0.4 GPa for stress. We demonstrate the predictive ability of the current SSW-NN method by exploring the global PES of a 48-atom TiO_2_ system with variable cell. This leads to the finding of two new porous TiO_2_ crystal phases, whose stabilities are comparable with that of the common TiO_2_ rutile phase. The solid-to-solid transition pathway sampling based on the global NN PES of TiO_2_ proves the kinetics stability of the porous structure.

For a single material, the computational cost for generating the global NN PES is up to a few hundreds of CPU cores for one week to compute the raw and the fine data set using quantum mechanics (DFT) calculations. In this work, in the TiO_2_ case, the actual computation time of DFT calculations for raw and fine data set generation is ∼0.94 × 10^6^ and ∼4.80 × 10^6^ seconds per CPU (24 cores), respectively (or ∼6.7 days for 10 CPUs in total). In applications of material discovery, the NN PES helps to greatly expedite the SSW global PES search. We estimate that the overall SSW-NN computational cost (NN PES construction plus SSW search = 1.3 × 10^7^ seconds per CPU) is roughly 10^3^ to 10^4^ times lower than that using SSW-DFT directly (10^10^ to 10^11^ seconds per CPU, see ESI Table S8†). Considering the automated process, perfect parallelization and generality of the SSW-NN method, we expect that it is time to build a comprehensive database of global NN PESs for different materials. With the collective efforts from the community, the global NN PESs can be further improved/tailored for specific applications that require a high accuracy in particularly local regions of PES. The SSW-NN method thus provides a promising solution to bridge the time-scale in material simulation and may open a new era for material discovery from theory.

## References

[cit1] Kohn W., Sham L. J. (1965). Phys. Rev..

[cit2] Wales D. J., Doye J. P. (1997). J. Phys. Chem. A.

[cit3] Doye J. P., Wales D. J. (1998). Phys. Rev. Lett..

[cit4] Wales D. J., Scheraga H. A. (1999). Science.

[cit5] Laio A., Parrinello M. (2002). Proc. Natl. Acad. Sci. U. S. A..

[cit6] Iannuzzi M., Laio A., Parrinello M. (2003). Phys. Rev. Lett..

[cit7] Deaven D. M., Ho K.-M. (1995). Phys. Rev. Lett..

[cit8] Woodley S., Battle P., Gale J., Catlow C. A. (1999). Phys. Chem. Chem. Phys..

[cit9] Turner G. W., Tedesco E., Harris K. D., Johnston R. L., Kariuki B. M. (2000). Chem. Phys. Lett..

[cit10] Oganov A. R., Glass C. W. (2006). J. Chem. Phys..

[cit11] Shang C., Liu Z. P. (2013). J. Chem. Theory Comput..

[cit12] Shang C., Zhang X. J., Liu Z. P. (2014). Phys. Chem. Chem. Phys..

[cit13] Behler J., Parrinello M. (2007). Phys. Rev. Lett..

[cit14] Artrith N., Morawietz T., Behler J. (2011). Phys. Rev. B: Condens. Matter Mater. Phys..

[cit15] Behler J. (2014). J. Phys.: Condens. Matter.

[cit16] Gastegger M., Marquetand P. (2015). J. Chem. Theory Comput..

[cit17] Shen X., Chen J., Zhang Z., Shao K., Zhang D. H. (2015). J. Chem. Phys..

[cit18] Shah S., Palmieri F., Datum M. (1992). Neural Network.

[cit19] Blank T. B., Brown S. D. (1994). J. Chemom..

[cit20] Hagan M. T., Menhaj M. B. (1994). IEEE Trans. Neural Network.

[cit21] Pukrittayakamee A., Malshe M., Hagan M., Raff L. M., Narulkar R., Bukkapatnum S., Komanduri R. (2009). J. Chem. Phys..

[cit22] Zhai H., Alexandrova A. N. (2016). J. Chem. Theory Comput..

[cit23] Shen L., Wu J., Yang W. (2016). J. Chem. Theory Comput..

[cit24] Behler J. (2011). J. Chem. Phys..

[cit25] Duane S., Kennedy A. D., Pendleton B. J., Roweth D. (1987). Phys. Lett. B.

[cit26] Clamp M. E., Baker P., Stirling C., Brass A. (1994). J. Comput. Chem..

[cit27] Williams D., Hinton G. (1986). Nature.

[cit28] Nocedal J. (1980). Math. Comput..

[cit29] Liu D. C., Nocedal J. (1989). Math. Program..

[cit30] Ngiam J., Coates A., Lahiri A., Prochnow B., Le Q. V. and Ng A. Y., Proceedings of the 28th International Conference on Machine Learning (ICML-11), 2011.

[cit31] Bottou L., Proceedings of Neuro-Nımes, 1991, vol. 91.

[cit32] Fletcher R., Reeves C. M. (1964). Comput. J..

[cit33] Polak E., Computational methods in optimization: a unified approach, Academic press, 1971.

[cit34] Chen J., Xu X., Xu X., Zhang D. H. (2013). J. Chem. Phys..

[cit35] Kirkpatrick S., Gelatt C. D., Vecchi M. P. (1983). science.

[cit36] Goedecker S. (2004). J. Chem. Phys..

[cit37] Zhang X. J., Shang C., Liu Z. P. (2013). J. Chem. Theory Comput..

[cit38] Zhai H.-J., Zhao Y.-F., Li W.-L., Chen Q., Bai H., Hu H.-S., Piazza Z. A., Tian W.-J., Lu H.-G., Wu Y.-B. (2014). Nat. Chem..

[cit39] Wei G.-F., Liu Z.-P. (2016). J. Chem. Theory Comput..

[cit40] Zhu S.-C., Xie S.-H., Liu Z.-P. (2015). J. Am. Chem. Soc..

[cit41] Guan S.-H., Zhang X.-J., Liu Z.-P. (2015). J. Am. Chem. Soc..

[cit42] Shang C., Liu Z.-P. (2012). J. Chem. Theory Comput..

[cit43] Zhao W.-N., Liu Z.-P. (2014). Chem. Sci..

[cit44] Zhao W.-N., Zhu S.-C., Li Y.-F., Liu Z.-P. (2015). Chem. Sci..

[cit45] Li Y.-F., Liu Z.-P., Liu L., Gao W. (2010). J. Am. Chem. Soc..

[cit46] Li Y.-F., Liu Z.-P. (2011). J. Am. Chem. Soc..

[cit47] Bakardjieva S., Stengl V., Szatmary L., Subrt J., Lukac J., Murafa N., Niznansky D., Cizek K., Jirkovsky J., Petrova N. (2006). J. Mater. Chem..

[cit48] Penn R. L., Banfield J. F. (1999). Am. Mineral..

[cit49] Cheng S., Wei-Na Z., Zhi-Pan L. (2015). J. Phys.: Condens. Matter.

[cit50] Kresse G., Hafner J. (1993). Phys. Rev. B: Condens. Matter Mater. Phys..

[cit51] Kresse G., Furthmüller J. (1996). Comput. Mater. Sci..

[cit52] Zhang X.-J., Shang C., Liu Z. (2017). Phys. Chem. Chem. Phys..

[cit53] Ewald P. P. (1921). Ann. Phys..

[cit54] Zhu S.-C., Xie S.-H., Liu Z.-P. (2014). J. Phys. Chem. Lett..

[cit55] Shang C., Liu Z.-P. (2010). J. Chem. Theory Comput..

[cit56] Zhang X. J., Liu Z. P. (2015). J. Chem. Theory Comput..

[cit57] Sasaki T., Watanabe M., Hashizume H., Yamada H., Nakazawa H. (1996). J. Am. Chem. Soc..

