# Supplementary material for: Material discovery by combining stochastic surface walking global optimization with a neural network
Source: Chem Sci. 2017 Jun 30;8(9):6327–37. doi: 10.1039/c7sc01459g (PMC5628601; doi:10.1039/c7sc01459g)
Supplement: Supplementary file 1 [file SC-008-C7SC01459G-s001.pdf]

Electronic Supplementary Material (ESI) for Chemical Science.
This journal is © The Royal Society of Chemistry 2017

Supporting information

Material Discovery by Combining Stochastic Surface Walking Global Optimization with Neural Network

*Si-da Huang, Cheng-Shang, Xiao-Jie Zhang, Zhi-Pan Liu**

*Collaborative Innovation Center of Chemistry for Energy Material, Shanghai Key Laboratory of Molecular Catalysis and Innovative Materials, Key Laboratory of Computational Physical Science (Ministry of Education), Department of Chemistry, Fudan University, Shanghai 200433, China *email: zpliu@fudan.edu.cn*

Table of Contents

1. **Derivation for the gradient of J_σ with respect to NN parameters**
2. **DFT calculation setups**
3. **Parameters of atom-centered symmetry functions for generating TiO_2 NN potential**
4. **Comparison of the performance for symmetry functions with different numbers of cutoff radii.**
5. **Comparison of the computational cost for NN and DFT calculations**
6. **Comparison of structural properties of experimentally known TiO_2 phase using NN and DFT PES.**
7. **XYZ coordination of structures.**

1. Derivation for the gradient of J_σ with respect to NN parameters

In the following, we derive the gradient of stress part of performance funciton J_σ with respect to NN parameters (see Figure 1 in main text for the NN architecture). In each subnet m (also network for element m), the value of node x in layer l, $a^{l,m}_x$, is constructed according to:

$$a^{l,m}_x = f^{l,m}\left(\sum_y w^{l,m}_{xy} a^{l-1,m}_y + b^{l,m}_x\right) \quad (S1)$$

$$z^{l,m}_x = \sum_y w^{l,m}_{xy} a^{l-1,m}_y + b^{l,m}_x \quad (S2)$$

where $w^{l,m}_{xy}$ is the weight of the subnet m that connects the node x in layer l and the node y in layer l-1; $b^{l,m}_i$ is the bias, and $f^{l,m}$ is the activation function. The stress part of performance function is written as:

$$J_\sigma = \frac{1}{18}\sum_{\alpha=1}^{3}\sum_{\beta=1}^{3}\left(\sigma^{NN}_{\alpha\beta} - \sigma^{real}_{\alpha\beta}\right)^2 \quad (S3)$$

$$\sigma^{NN}_{\alpha\beta} = -\frac{1}{V}\sum_{m=1}^{N_{ele}}\sum_{i=1}^{N_{atm,m}}\sum_{j=1}^{M_{i,m}}\sum_{d=1}^{D_{j,i,m}} \frac{(r_d)_\alpha (r_d)_\beta}{r_d}\frac{\partial E^m_i}{\partial G_{j,i,m}}\frac{\partial G_{j,i,m}}{\partial r_d} \quad (S4)$$

where $\sigma^{NN}_{\alpha\beta}$, $\sigma^{real}_{\alpha\beta}$ are the stress tensor matrix element in NN PES and DFT, respectively; $G_{j,i,m}$ is the j-th symmetry function describing atom i of element m; r_d is the distance vector with the module r_d constituing the symmtry function; V is the volume of structure.

Combinging Eqs. (S3-4) with Eqs. (S1-2), the gradient for the stress part of performance function with respect to a network weight and bias follows the expression:

$$\frac{\partial J_\sigma}{\partial w^{l,m}_{xy}} = -\frac{1}{9V}\sum_{\alpha=1}^{3}\sum_{\beta=1}^{3}\left[\left(\sigma^{NN}_{\alpha\beta} - \sigma^{real}_{\alpha\beta}\right)\sum_{m=1}^{N_{ele}}\sum_{i=1}^{N_{atm,m}}\sum_{j=1}^{M_{i,m}}\sum_{d=1}^{D_{j,i,m}} \frac{(r_d)_\alpha (r_d)_\beta \partial G_{j,i,m}}{r_d \quad \partial r_d}\frac{\partial}{\partial w^{l,m}_{xy}}\frac{\partial E^m_i}{\partial G_{j,i,m}}\right] \quad (S5)$$

$$\frac{\partial J_\sigma}{\partial b^{l,m}_{x}} = -\frac{1}{9V}\sum_{\alpha=1}^{3}\sum_{\beta=1}^{3}\left[\left(\sigma^{NN}_{\alpha\beta} - \sigma^{real}_{\alpha\beta}\right)\sum_{m=1}^{N_{ele}}\sum_{i=1}^{N_{atm,m}}\sum_{j=1}^{M_{i,m}}\sum_{d=1}^{D_{j,i,m}} \frac{(r_d)_\alpha (r_d)_\beta \partial G_{j,i,m}}{r_d \quad \partial r_d}\frac{\partial}{\partial b^{l,m}_{x}}\frac{\partial E^m_i}{\partial G_{j,i,m}}\right] \quad (S6)$$

For simplicity, we define the following terms:

$$fac_{j,i,m} = -\frac{1}{9V}\sum_{\alpha=1}^{3}\sum_{\beta=1}^{3}\sum_{d=1}^{D_{j,i,m}}\left(\sigma^{NN}_{\alpha\beta} - \sigma^{real}_{\alpha\beta}\right)\frac{(r_d)_\alpha (r_d)_\beta \partial G_{j,i,m}}{r_d \quad \partial r_d} \quad (S7)$$

$$u_{i,j,x}^{l,m} = fac_{j,i,m}\frac{\partial E_i^m}{\partial z_{i,x}^{l,m}} \text{ (S8)}$$

$$v_{i,x}^{l,m} = \sum_{j=1}^{M_{i,m}} fac_{j,i,m}\frac{\partial}{\partial G_{j,i,m}}\frac{\partial E_i^m}{\partial z_{i,x}^{l,m}} \text{ (S9)}$$

to reduce Eqs. S5-6 as:

$$\frac{\partial J_\sigma}{\partial w_{xy}^{l,m}} = \sum_{i=1}^{N_{atm,m}}\sum_{j=1}^{M_{i,m}}\frac{\partial a_{i,y}^{l-1,m}}{\partial G_{j,i,m}}u_{i,j,x}^{l,m} + \sum_{i=1}^{N_{atm,m}} a_{i,y}^{l-1,m}v_{i,x}^{l,m} \text{ (S10)}$$

$$\frac{\partial J_\sigma}{\partial b_x^{l,m}} = \sum_{i=1}^{N_{atm,m}} v_{i,x}^{l,m} \text{ (S11)}$$

To get the derivatives in Eqs. S10-11, we need to compute the following three terms $\frac{\partial a_{i,x}^{l,m}}{\partial G_{j,i,m}}$, $u_{i,j,x}^{l,m}$ and $v_{i,x}^{l,m}$,. The first term is updated forwardly from layer to layer using:

$$\frac{\partial a_{i,x}^{l,m}}{\partial G_{j,i,m}} = f'\left(z_{i,x}^{l,m}\right)\sum_{k=1}^{S_{l-1}} w_{xk}^{l,m}\frac{\partial a_{i,k}^{l-1,m}}{\partial G_{j,i,m}} \text{ (S12)}$$

which is initialized at the first layer with

$$\frac{\partial a_{i,x}^{l,m}}{\partial G_{j,i,m}} = \begin{cases} 1, & x = j \\ 0, & x \neq j \end{cases} \text{ (S13)}$$

The other two terms $u_{i,j,x}^{l,m}$ and $v_{i,x}^{l,m}$ are updated back-propagatedly from layer to layer using:

$$u_{i,j,x}^{l,m} = f'\left(z_{i,x}^{l,m}\right)\sum_{k=1}^{S_{l+1}} w_{kx}^{l+1,m}u_{i,j,k}^{l+1,m} \text{ (S14)}$$

$$v_{i,x}^{l,m} = f'\left(z_{i,x}^{l,m}\right)\sum_{k=1}^{S_{l+1}} w_{kx}^{l+1,m}v_{i,j,k}^{l+1,m} + \sum_{j=1}^{M_{i,m}}\frac{\partial f'\left(z_{i,x}^{l,m}\right)}{\partial G_{j,i,m}}\sum_{k=1}^{S_{l+1}} w_{kx}^{l+1,m}u_{i,j,k}^{l+1,m} \text{ (S15)}$$

which is initialized at the last layer (layer L) using:

$$u_{i,j,x}^{L,m} = fac_{j,i,m} \cdot f'\left(z_{i,x}^{L,m}\right) \text{ (S16)}$$

$$v_{i,x}^{L,m} = \sum_{j=1}^{M_{i,m}} fac_{j,i,m} \cdot \frac{\partial f'\left(z_{i,x}^{L,m}\right)}{\partial G_{j,i,m}} \text{ (S17)}$$

2. DFT calculation setups

All DFT calculations are performed using the periodic plane wave method as implemented in the VASP package[1-2]. The ionic core electrons are described using the projector augmented wave (PAW) pseudopotential[3], where the semicore electrons are included in the Ti potential ($3p^6 3d^2 4s^2$) .The electron exchange and correlation effects are described by the GGA-PBE functional[4]. For the low accuracy calculations used in SSW sampling to collect raw data set (Scheme 1 in text), the kinetic energy cutoff utilized is 400 eV; the first Brillouin zone is sampled using the Monkhorst−Pack scheme[5] with a (4 × 4 × 4). For the high accuracy calculations used in single-point energy refinement calculations to build the global data set, these two quantities increase to 600 eV and (6 × 6 × 6), respectively.

(1) Kresse, G.; Hafner, J., *Physical Review B* **1993,** *47*, 558.

(2) Kresse, G.; Furthmüller, J., *Computational Materials Science* **1996,** *6*, 15-50.

(3) Kresse, G.; Joubert, D., *Physical Review B* **1999,** *59*, 1758.

(4) Perdew, J. P.; Burke, K.; Ernzerhof, M., *Physical review letters* **1996,** *77*, 3865.

(5) Monkhorst, H. J.; Pack, J. D., *Physical review B* **1976,** *13*, 5188.

3. Parameters of atom-centered symmetry functions for generating TiO_2 NN potential

Table S1. Parameters of the radial symmetry functions G^1 (see Eq. 3 in the main text) used to describe the local atomic environment ('Cent ele' in table). R_c is the cutoff radius, and 'Neigb ele' is the neighboring element taken into account.

Cent ele	No.	R_c (Å)	Neigb ele	Cent ele	No.	R_c (Å)	Neigb ele
O	1	3.30	O	O	3	6.00	O
O	2	3.30	Ti	O	4	6.00	Ti
Ti	1	3.30	O	Ti	3	6.00	O
Ti	2	3.30	Ti	Ti	4	6.00	Ti

Table S2. Parameters of the radial symmetry functions G^2 (see Eq. 4 in main text) used to describe the local atomic environment. Also see Table S1 caption for explanations.

Cent ele	R_c (Å)	No.	Neigb ele	η (Å^{-2})	R_s (Å)	Cent ele	R_c (Å)	No.	Neigb ele	η (Å^{-2})	R_s (Å)
O	3.30	1	O	0.042	1.8	O	6.00	9	O	0.014	2.0
O	3.30	2	O	0.280	0.2	O	6.00	10	O	0.028	1.0
O	3.30	3	O	0.280	0.5	O	6.00	11	O	0.140	0.5
O	3.30	4	O	0.560	1.0	O	6.00	12	O	0.504	1.8
O	3.30	5	Ti	0.042	2.0	O	6.00	13	Ti	0.014	2.0
O	3.30	6	Ti	0.280	0.2	O	6.00	14	Ti	0.028	1.0
O	3.30	7	Ti	0.280	0.5	O	6.00	15	Ti	0.140	0.5
O	3.30	8	Ti	0.560	1.0	O	6.00	16	Ti	0.504	1.8
Ti	3.30	1	O	0.042	2.0	Ti	6.00	9	O	0.014	2.0
Ti	3.30	2	O	0.280	0.2	Ti	6.00	10	O	0.028	1.0
Ti	3.30	3	O	0.280	0.5	Ti	6.00	11	O	0.140	0.5
Ti	3.30	4	O	0.560	1.0	Ti	6.00	12	O	0.504	1.8
Ti	3.30	5	Ti	0.042	2.2	Ti	6.00	13	Ti	0.014	2.2
Ti	3.30	6	Ti	0.280	0.2	Ti	6.00	14	Ti	0.028	1.0
Ti	3.30	7	Ti	0.280	0.5	Ti	6.00	15	Ti	0.140	0.5
Ti	3.30	8	Ti	0.560	1.0	Ti	6.00	16	Ti	0.504	1.8

Table S3. Parameters of the radial symmetry functions G^3 (see Eq. 5 in main text) used to describe the local atomic environments. Also see Table S1 caption for explanations.

Cent ele	R_c (Å)	No.	Neigb ele	κ ($Å^{-1}$)	Cent ele	R_c (Å)	No.	Neigb ele	κ ($Å^{-1}$)
O	3.30	1	O	0.265	O	3.30	4	Ti	0.794
O	3.30	2	O	0.794	O	6.00	5	O	0.529
O	3.30	3	Ti	0.265	O	6.00	6	Ti	0.529
Ti	3.30	1	O	0.265	Ti	3.30	4	Ti	0.794
Ti	3.30	2	O	0.794	Ti	6.00	5	O	0.529
Ti	3.30	3	Ti	0.265	Ti	6.00	6	Ti	0.529

Table S4. Parameters of the radial symmetry functions G^4 (see Eq. 6 in main text) used to describe the local atomic environment. Also see Table S1 caption for explanations.

Cent ele	R_c (Å)	No.	Neigb ele1	Neigb ele2	η (Å^{-2})	λ	ζ	Cent ele	R_c (Å)	No.	Neigb ele1	Neigb ele2	η (Å^{-2})	λ	ζ
O	3.30	1	O	O	0.000	1.0	-1.0	O	3.30	16	Ti	Ti	0.011	4.0	1.0
O	3.30	2	O	O	0.000	1.0	1.0	O	3.30	17	Ti	Ti	0.022	1.0	-1.0
O	3.30	3	O	O	0.011	1.0	-1.0	O	3.30	18	Ti	Ti	0.022	1.0	1.0
O	3.30	4	O	O	0.011	4.0	1.0	O	6.00	19	O	O	0.003	1.0	-1.0
O	3.30	5	O	O	0.022	1.0	-1.0	O	6.00	20	O	O	0.003	4.0	1.0
O	3.30	6	O	O	0.022	1.0	1.0	O	6.00	21	O	O	0.014	1.0	-1.0
O	3.30	7	O	Ti	0.000	1.0	-1.0	O	6.00	22	O	O	0.014	2.0	1.0
O	3.30	8	O	Ti	0.000	1.0	1.0	O	6.00	23	O	Ti	0.003	1.0	-1.0
O	3.30	9	O	Ti	0.011	1.0	-1.0	O	6.00	24	O	Ti	0.003	4.0	1.0
O	3.30	10	O	Ti	0.011	4.0	1.0	O	6.00	25	O	Ti	0.014	1.0	-1.0
O	3.30	11	O	Ti	0.022	1.0	-1.0	O	6.00	26	O	Ti	0.014	2.0	1.0
O	3.30	12	O	Ti	0.022	4.0	1.0	O	6.00	27	Ti	Ti	0.003	1.0	-1.0
O	3.30	13	Ti	Ti	0.000	1.0	-1.0	O	6.00	28	Ti	Ti	0.003	4.0	1.0
O	3.30	14	Ti	Ti	0.000	1.0	1.0	O	6.00	29	Ti	Ti	0.014	1.0	-1.0
O	3.30	15	Ti	Ti	0.011	1.0	-1.0	O	6.00	30	Ti	Ti	0.014	2.0	1.0
Ti	3.30	1	O	O	0.000	1.0	-1.0	Ti	6.00	13	O	O	0.003	1.0	-1.0
Ti	3.30	2	O	O	0.000	1.0	1.0	Ti	6.00	14	O	O	0.003	4.0	1.0
Ti	3.30	3	O	O	0.011	1.0	-1.0	Ti	6.00	15	O	O	0.014	1.0	-1.0
Ti	3.30	4	O	O	0.011	4.0	1.0	Ti	6.00	16	O	O	0.014	2.0	1.0
Ti	3.30	5	O	O	0.022	1.0	-1.0	Ti	6.00	17	O	Ti	0.003	1.0	-1.0
Ti	3.30	6	O	O	0.022	1.0	1.0	Ti	6.00	18	O	Ti	0.003	4.0	1.0
Ti	3.30	7	O	Ti	0.000	1.0	-1.0	Ti	6.00	19	O	Ti	0.014	1.0	-1.0
Ti	3.30	8	O	Ti	0.000	1.0	1.0	Ti	6.00	20	O	Ti	0.014	2.0	1.0
Ti	3.30	9	O	Ti	0.011	1.0	-1.0	Ti	6.00	21	Ti	Ti	0.003	1.0	-1.0
Ti	3.30	10	O	Ti	0.011	4.0	1.0	Ti	6.00	22	Ti	Ti	0.003	4.0	1.0
Ti	3.30	11	O	Ti	0.022	1.0	-1.0	Ti	6.00	23	Ti	Ti	0.014	1.0	-1.0
Ti	3.30	12	O	Ti	0.022	1.0	1.0	Ti	6.00	24	Ti	Ti	0.014	2.0	1.0

Table S5. Parameters of the radial symmetry functions G^5 (see Eq. 7 in main text) used to describe the local atomic environment. Also see Table 1 caption for explanations.

Cent ele	R_c (Å)	No.	Neigb ele1	Neigb ele2	η (Å^{-2})	λ	ζ	Cent ele	R_c (Å)	No.	Neigb ele1	Neigb ele2	η (Å^{-2})	λ	ζ
O	3.30	1	O	O	0.112	1.0	-1.0	O	6.00	16	O	O	0.140	4.0	1.0
O	3.30	2	O	O	0.112	4.0	1.0	O	6.00	17	O	O	0.196	1.0	1.0
O	3.30	3	O	O	0.168	1.0	-1.0	O	6.00	18	O	O	0.196	4.0	-1.0
O	3.30	4	O	O	0.224	1.0	1.0	O	6.00	19	O	Ti	0.007	1.0	-1.0
O	3.30	5	O	Ti	0.112	1.0	-1.0	O	6.00	20	O	Ti	0.007	2.0	1.0
O	3.30	6	O	Ti	0.112	4.0	1.0	O	6.00	21	O	Ti	0.140	1.0	-1.0
O	3.30	7	O	Ti	0.168	1.0	-1.0	O	6.00	22	O	Ti	0.140	4.0	1.0
O	3.30	8	O	Ti	0.224	1.0	1.0	O	6.00	23	O	Ti	0.196	1.0	1.0
O	3.30	9	Ti	Ti	0.112	1.0	-1.0	O	6.00	24	O	Ti	0.196	4.0	-1.0
O	3.30	10	Ti	Ti	0.112	4.0	1.0	O	6.00	25	Ti	Ti	0.007	1.0	-1.0
O	3.30	11	Ti	Ti	0.168	1.0	-1.0	O	6.00	26	Ti	Ti	0.007	2.0	1.0
O	3.30	12	Ti	Ti	0.224	1.0	1.0	O	6.00	27	Ti	Ti	0.140	1.0	-1.0
O	6.00	13	O	O	0.007	1.0	-1.0	O	6.00	28	Ti	Ti	0.140	4.0	1.0
O	6.00	14	O	O	0.007	2.0	1.0	O	6.00	29	Ti	Ti	0.196	1.0	1.0
O	6.00	15	O	O	0.140	1.0	-1.0	O	6.00	30	Ti	Ti	0.196	4.0	-1.0
Ti	3.30	1	O	O	0.112	1.0	-1.0	Ti	6.00	16	O	O	0.140	4.0	1.0
Ti	3.30	2	O	O	0.112	4.0	1.0	Ti	6.00	17	O	O	0.196	1.0	1.0
Ti	3.30	3	O	O	0.168	1.0	-1.0	Ti	6.00	18	O	O	0.196	4.0	-1.0
Ti	3.30	4	O	O	0.224	1.0	1.0	Ti	6.00	19	O	Ti	0.007	1.0	-1.0
Ti	3.30	5	O	Ti	0.112	1.0	-1.0	Ti	6.00	20	O	Ti	0.007	2.0	1.0
Ti	3.30	6	O	Ti	0.112	4.0	1.0	Ti	6.00	21	O	Ti	0.140	1.0	-1.0
Ti	3.30	7	O	Ti	0.168	1.0	-1.0	Ti	6.00	22	O	Ti	0.140	4.0	1.0
Ti	3.30	8	O	Ti	0.224	1.0	1.0	Ti	6.00	23	O	Ti	0.196	1.0	1.0
Ti	3.30	9	Ti	Ti	0.112	1.0	-1.0	Ti	6.00	24	O	Ti	0.196	4.0	-1.0
Ti	3.30	10	Ti	Ti	0.112	4.0	1.0	Ti	6.00	25	Ti	Ti	0.007	1.0	-1.0
Ti	3.30	11	Ti	Ti	0.168	1.0	-1.0	Ti	6.00	26	Ti	Ti	0.007	2.0	1.0
Ti	3.30	12	Ti	Ti	0.224	1.0	1.0	Ti	6.00	27	Ti	Ti	0.140	1.0	-1.0
Ti	6.00	13	O	O	0.007	1.0	-1.0	Ti	6.00	28	Ti	Ti	0.140	4.0	1.0
Ti	6.00	14	O	O	0.007	2.0	1.0	Ti	6.00	29	Ti	Ti	0.196	1.0	1.0
Ti	6.00	15	O	O	0.140	1.0	-1.0	Ti	6.00	30	Ti	Ti	0.196	4.0	-1.0

4. Comparison of the performance for symmetry functions with different numbers of cutoff radii.

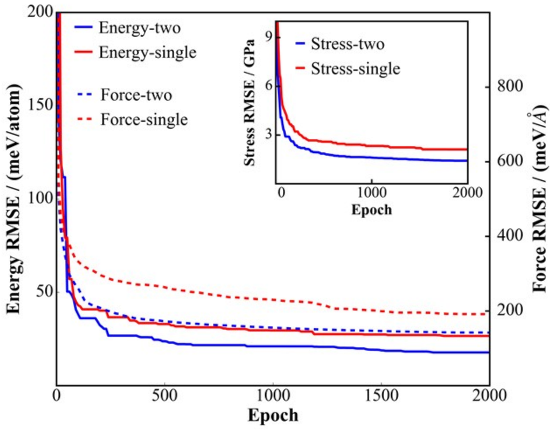

Figure S1. Comparison of the performance for symmetry functions with 2-cutoffs (blue) and single-cutoff (red) radius (R_c in Eq. 2 of main text) by showing the evolution of RMSEs of energy (meV / atom) and force (meV / Å) in the training set during the NN training. 2-cutoffs symmetry functions correspond to Table S1-S5 NN parameters; 1-cutoff symmetry functions correspond to Table S1-S5 NN parameters except all R_c=3.3 are replaced by R_c=6.0. Solid lines: RMSEs of energy; Dashed lines: RMSEs of forces. The inset is the comparison for the stress.

5. Comparison of the computational cost for NN and DFT calculations.

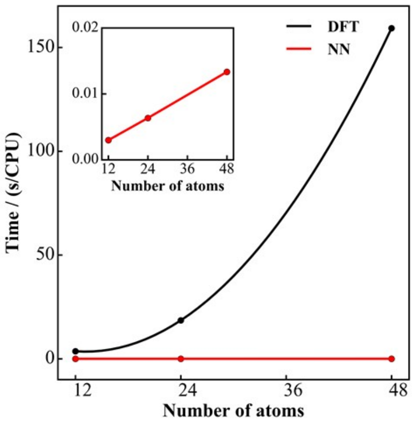

Figure S2. Computational time (s/CPU) of single point calculation for different size TiO_2 systems (12, 24 and 48 atoms in a unit cell) by DFT (black) and NN (red). The inset rescales y-axis to illustrate the NN performance better. Also see Table S7 for the data.

Table S7. Computation time (seconds per CPU, each CPU contains 24 cores) in a single point energy calculation for different TiO_2 systems (12, 24 and 48 atoms in one unit cell) by DFT and by NN.

TiO_2	T_{low}*	T_{high}*	T_{NN}*	S_{low}‡	S_{high}‡
12-atom	7.2	96.0	0.13	55	738
24-atom	62.4	672.0	0.26	240	2584
48-atom	631.2	8205.6	0.39	1618	21040

* T_{low}: time using low precision DFT ((Ti PAW potential with 4 electrons (Ti: $3d^2 4s^2$); (4×4×4) k-points and 400 eV cutoff); T_{high}: time using high precision DFT (Ti PAW potential with 10 electrons (Ti: $3p^6 3d^2 4s^2$); (6×6×6) k-points and 600 eV cutoff); $T_{NN:}$ time using NN calculations that utilizes only one CPU core per run.

‡ S_{low}/S_{high}: The scaling of NN with respect to low/high precision DFT.

Table S8 shows that tthe overall cost of SSW-NN for the global PES search of TiO_2 in 48-atom cell is still roughly four orders of magnitude lower than that of SSW-DFT.

Table S8. Computation time (10^6 seconds per CPU, each CPU contains 24 cores) of SSW global search for TiO_2 in 48-atom cell.

	SSW-NN	SSW-DFT_{low}	SSW-DFT_{high}
Comput. time	12.76 (0.94/4.80/7.02)	$1.14*10^4$	$1.48*10^5$

* The overall computational cost in SSW-NN is contributed from three sources, (i) SSW global search using low-precision DFT in 12-atom cell, which collects 130,000 structures from SSW trajectories; (ii) the high-precision DFT single-point calculations to construct training data set (50,000 structures); and (iii) SSW global search in 48-atom cell ($1.8*10^7$ energy/force evaluations to glean 60,000 minima). The data in parenthesis list the computation time for each part.

** The computational cost in SSW-DFT-low/high is estimated from SSW global search in 48-atom cell ($1.8*10^7$ energy/force evaluations) to identify 60,000 minima with the low/high DFT accuracy setups (also see Table S7).

6. Comparison of structural properties of experimentally known TiO_2 phase using NN and DFT PES.

Table S9. Comparison of the energy and lattice parameters of experimentally known structures of TiO_2 using NN PES and DFT calculations. All energies are with reference to that of GM (TiO_2-B).

No	Species	SG*	OP_2*	E_{DFT}/eV/f.u.	E_{NN}/eV/f.u.	dE**/eV/f.u.	V_{DFT}/Å^3/f.u.	V_{NN}/Å^3/f.u.	dV**/Å^3/f.u.
1	rutile	136	0.28	0.11	0.11	0.00	32.15	32.20	0.05
2	anatase	141	0.37	0.02	0.01	-0.01	35.19	35.17	-0.02
3	brookite	61	0.30	0.05	0.05	0.00	33.06	33.07	0.01
4	TiO_2(B)	12	0.52	0.00	-0.01	-0.01	36.66	36.82	0.16
5	TiO_2(H)	87	0.65	0.18	0.22	0.04	39.56	39.89	0.33
6	TiO_2(R)	62	0.34	0.18	0.19	0.01	35.31	35.69	0.38
7	TiO_2(II)	60	0.23	0.09	0.10	0.01	31.54	31.72	0.18
8	baddeleyite	14	0.18	0.20	0.21	0.01	29.89	29.91	0.02
9	TiO_2-OI	61	0.14	0.35	0.36	0.01	28.50	28.65	0.15
10	TiO_2-OII	62	0.02	0.87	0.85	-0.02	26.25	26.35	0.10

*SG: symmetry group; OP_2: Steinhardt-type order parameter (see Eq. 11);

** dE and dV: deviation of energy and volume from NN PES with respect to those (E_{DFT} and V_{DFT}) from DFT PES, respectively.

7. XYZ coordination of structures.

```
        TiO2-B        Energy from VASP is -107.69
!DATE
PBC     6.4304      3.7628      6.6265      89.9908   106.2060     72.9920
O     0.583206356     1.842667707     0.611031485     CORE     1    O    O    0.0000     1
O     4.852912827     0.536925427     5.001861099     CORE     2    O    O    0.0000     2
O     3.267794823     2.989255309     5.149575118     CORE     3    O    O    0.0000     3
O     3.268834054     2.988334428     0.463463701     CORE     4    O    O    0.0000     4
O     4.822747234     2.512947509     2.829482641     CORE     5    O    O    0.0000     5
O     0.545506551     1.854904490     4.666370234     CORE     6    O    O    0.0000     6
O     1.713743047     3.464882796     2.783458911     CORE     7    O    O    0.0000     7
O     4.890581050     0.524616285     0.946684241     CORE     8    O    O    0.0000     8
Ti    5.030237067     2.450060374     4.595621732     CORE     9    Ti   Ti   0.0000     9
Ti    1.549049436     3.515142833     4.644133649     CORE     10   Ti   Ti   0.0000     10
Ti    1.506476318     3.527856727     1.017392971     CORE     11   Ti   Ti   0.0000     11
Ti    4.987475916     2.462268399     0.968835576     CORE     12   Ti   Ti   0.0000     12
end
end
```

```
   anatase        Energy from VASP is -107.62
!DATE
PBC     5.3814      5.3813      5.5546    118.9567      89.9897      89.9978
O     4.194198606     0.856082428     2.579820839     CORE     1    O    O    0.0000     1
O     0.158673073     4.892198289     0.149748701     CORE     2    O    O    0.0000     2
O     1.503583919     3.546716138     2.579809188     CORE     3    O    O    0.0000     3
O     4.194514209     3.546897938     1.733259159     CORE     4    O    O    0.0000     4
O     2.849294535     2.201561852     0.149734903     CORE     5    O    O    0.0000     5
O     2.850637267     2.203090838     4.163341984     CORE     6    O    O    0.0000     6
O     1.503713032     0.856256749     1.733261386     CORE     7    O    O    0.0000     7
O     0.159823349    -0.487541830     4.163349920     CORE     8    O    O    0.0000     8
Ti    2.848871027     2.201289809     2.156503676     CORE     9    Ti   Ti   0.0000     9
Ti    4.196250059     0.857770012     4.586677458     CORE     10   Ti   Ti   0.0000     10
Ti    0.158051496    -0.489353156     2.156505204     CORE     11   Ti   Ti   0.0000     11
Ti    1.505462630    -1.832849123     4.586689365     CORE     12   Ti   Ti   0.0000     12
end
end
```

```
   TiO2-II        Energy from VASP is -107.32
!DATE
PBC     5.5782      4.5845      4.9336      89.9986      90.0000      89.9982
O     2.655829461     4.573045084     3.201128877     CORE     1    O    O    0.0000     1
O     3.987086485     4.573020961     0.734325392     CORE     2    O    O    0.0000     2
O     1.197925484     2.280816988     4.003218636     CORE     3    O    O    0.0000     3
O     5.444742859     2.280728980     1.536376872     CORE     4    O    O    0.0000     4
O     3.987017536     2.085187894     4.003065820     CORE     5    O    O    0.0000     5
O     2.655611840     2.085119376     1.536287472     CORE     6    O    O    0.0000     6
O     5.444867602     4.377405597     3.201102411     CORE     7    O    O    0.0000     7
O     1.197990440     4.377370075     0.734361893     CORE     8    O    O    0.0000     8
Ti    2.336869024     1.036666902     4.835611361     CORE     9    Ti   Ti   0.0000     9
Ti    5.125943791     3.328912914     4.835518682     CORE     10   Ti   Ti   0.0000     10
Ti    4.305828900     1.036696731     2.368706908     CORE     11   Ti   Ti   0.0000     11
Ti    1.516894587     3.328971422     2.368821752     CORE     12   Ti   Ti   0.0000     12
end
end
```

```
   rutile        Energy from VASP is -107.25
```

```
!DATE
PBC    5.5231    5.5229    4.6491    90.0127    89.9874    114.9372
O    3.206710125    4.875312726    4.082738586    CORE    1    O    O    0.0000    1
O    3.135355807    1.398276571    2.264141858    CORE    2    O    O    0.0000    2
O    1.971011238    3.902335742    1.763171760    CORE    3    O    O    0.0000    3
O    1.609429377    2.371291940    4.082724135    CORE    4    O    O    0.0000    4
O    4.371392056    2.371015026    4.593864082    CORE    5    O    O    0.0000    5
O    0.445521159    4.875053112    4.593852397    CORE    6    O    O    0.0000    6
O    0.373751649    1.398335351    1.763174150    CORE    7    O    O    0.0000    7
O    -0.790550723    3.902325833    2.264134949    CORE    8    O    O    0.0000    8
Ti    3.120578828    3.166075662    3.162235067    CORE    9    Ti    Ti    0.0000    9
Ti    0.359450991    3.165707320    0.860251469    CORE    10    Ti    Ti    0.0000    10
Ti    4.285121573    0.661759475    0.859772344    CORE    11    Ti    Ti    0.0000    11
Ti    1.523354732    0.662112520    3.162098123    CORE    12    Ti    Ti    0.0000    12
end
end
```

Str-1   Energy from VASP is -107.20

```
!DATE
PBC    8.0611    8.0631    5.9485    68.4633    111.6457    92.8532
O    -1.302320157    5.040501508    4.137228810    CORE    1    O    O    0.0000    1
O    -0.195070727    3.994643706    1.582453061    CORE    2    O    O    0.0000    2
O    1.268578344    3.506011922    4.149575341    CORE    3    O    O    0.0000    3
O    -1.330465840    2.143301850    3.585507681    CORE    4    O    O    0.0000    4
O    4.933092024    0.930782837    1.587825913    CORE    5    O    O    0.0000    5
O    3.838716495    1.968275715    4.151339823    CORE    6    O    O    0.0000    6
O    5.098199777    7.531279249    4.149822886    CORE    7    O    O    0.0000    7
O    3.869874762    4.863501881    4.711176136    CORE    8    O    O    0.0000    8
Ti    4.843603654    9.159627174    3.375242474    CORE    9    Ti    Ti    0.0000    9
Ti    2.906385832    3.329820636    4.925161378    CORE    10    Ti    Ti    0.0000    10
Ti    -0.367576692    3.677321107    3.369516885    CORE    11    Ti    Ti    0.0000    11
Ti    5.355308420    5.900904752    4.919529377    CORE    12    Ti    Ti    0.0000    12
end
end
```

Str-2   Energy from VASP is -107.14

```
!DATE
PBC    5.8993    6.8748    6.1557    102.3379    117.9044    93.7558
O    1.801955829    3.569277669    0.060420798    CORE    1    O    O    0.0000    1
O    -0.191671612    5.225018718    1.282291004    CORE    2    O    O    0.0000    2
O    -2.070751520    4.748257556    3.850519296    CORE    3    O    O    0.0000    3
O    -0.077009894    3.092450350    2.629024480    CORE    4    O    O    0.0000    4
O    0.906188426    4.887035630    4.437929689    CORE    5    O    O    0.0000    5
O    0.232094015    1.299884064    0.574971091    CORE    6    O    O    0.0000    6
O    -0.050869794    0.157576473    3.335994357    CORE    7    O    O    0.0000    7
O    1.843594573    1.923928726    4.699894788    CORE    8    O    O    0.0000    8
Ti    -0.423023291    5.167385441    3.200288158    CORE    9    Ti    Ti    0.0000    9
Ti    2.576905751    4.991864681    5.156494505    CORE    10    Ti    Ti    0.0000    10
Ti    0.154172001    3.150187049    0.710730356    CORE    11    Ti    Ti    0.0000    11
Ti    0.172825678    1.818389519    3.981545837    CORE    12    Ti    Ti    0.0000    12
end
end
```

lepidocrocite   Energy from VASP is -106.99

```
!DATE
PBC    4.8158    4.8158    9.2595    58.5131    96.2773    102.1620
O    1.322927203    5.796281737    3.577153131    CORE    1    O    O    0.0000    1
O    1.322872063    5.796766856    6.240600409    CORE    2    O    O    0.0000    2
```

O -1.085077270 5.796434527 1.931730499 CORE 3 O O 0.0000 3
O 1.830312750 3.442532548 4.595204295 CORE 4 O O 0.0000 4
O -1.084860546 5.796410092 4.595204295 CORE 5 O O 0.0000 5
O -1.592301233 8.150644399 6.240600409 CORE 6 O O 0.0000 6
O 1.830096026 3.442556984 1.931730500 CORE 7 O O 0.0000 7
O -0.577657656 3.442404193 3.577153130 CORE 8 O O 0.0000 8
Ti 0.372833114 4.619615543 5.205280234 CORE 9 Ti Ti 0.0000 9
Ti 2.780490140 4.619381317 2.967076083 CORE 10 Ti Ti 0.0000 10
Ti 2.273417972 6.973493087 5.205280234 CORE 11 Ti Ti 0.0000 11
Ti 0.879905282 2.265503774 2.967076082 CORE 12 Ti Ti 0.0000 12
end
end

TiO2-R Energy from VASP is -106.98
!DATE
PBC 9.5346 2.9807 4.9697 90.0000 89.9874 90.0000
O 9.250472601 0.745186196 3.955928451 CORE 1 O O 0.0000 1
O 2.543027823 0.745185938 1.820819256 CORE 2 O O 0.0000 2
O 5.053269320 2.235515369 1.471344893 CORE 3 O O 0.0000 3
O 2.225413683 2.235514994 4.305858904 CORE 4 O O 0.0000 4
O 0.285213295 2.235515242 1.013718398 CORE 5 O O 0.0000 5
O 6.992711083 2.235515024 3.148735299 CORE 6 O O 0.0000 6
O 4.482492335 0.745185796 3.498364500 CORE 7 O O 0.0000 7
O 7.310159186 0.745185664 0.663745731 CORE 8 O O 0.0000 8
Ti 1.312515779 0.745186028 0.398085138 CORE 9 Ti Ti 0.0000 9
Ti 3.455318003 2.235515784 2.882509640 CORE 10 Ti Ti 0.0000 10
Ti 8.223160766 2.235515853 4.571564302 CORE 11 Ti Ti 0.0000 11
Ti 6.080341397 0.745186182 2.087224789 CORE 12 Ti Ti 0.0000 12
end
end

TiO2-H Energy from VASP is -106.97
!DATE
PBC 7.4357 7.4367 2.9812 78.4418 101.5642 92.3150
O 2.301270862 7.153607639 0.598271524 CORE 1 O O 0.0000 1
O 5.033741933 2.908725785 0.467579271 CORE 2 O O 0.0000 2
O 4.236413917 0.850632071 2.265509473 CORE 3 O O 0.0000 3
O 1.503947385 5.095569340 2.396193876 CORE 4 O O 0.0000 4
O 6.755523488 4.811372539 0.445757690 CORE 5 O O 0.0000 5
O 2.403391580 2.499104005 1.552667186 CORE 6 O O 0.0000 6
O -0.217821413 3.192921995 2.418009631 CORE 7 O O 0.0000 7
O 4.134231022 5.505209580 1.311084134 CORE 8 O O 0.0000 8
Ti 3.830110043 1.470289167 0.505210106 CORE 9 Ti Ti 0.0000 9
Ti 2.707567933 6.533979771 2.358563092 CORE 10 Ti Ti 0.0000 10
Ti 1.015337433 3.648749000 1.032638809 CORE 11 Ti Ti 0.0000 11
Ti 5.522360072 4.355517238 1.831132974 CORE 12 Ti Ti 0.0000 12
end
end

baddeleyite Energy from VASP is -106.91
!DATE
PBC 4.8534 5.0956 4.9099 89.9962 90.0001 100.0987
O 0.092611133 0.124278447 4.231390402 CORE 1 O O 0.0000 1
O 2.724459787 2.093200823 2.166300671 CORE 2 O O 0.0000 2
O 2.277789540 4.601478183 2.085133135 CORE 3 O O 0.0000 3
O 2.286959669 1.703460486 4.540088916 CORE 4 O O 0.0000 4
O -0.354166690 2.632313847 0.019959797 CORE 5 O O 0.0000 5
O 1.840153772 4.211787267 4.621178328 CORE 6 O O 0.0000 6

```
O     0.512243252     1.163912441     1.776383844     CORE     7     O     O     0.0000     7
O     0.065435937     3.672271260     2.474995621     CORE     8     O     O     0.0000     8
Ti    3.335423032     3.323631682     3.629101341     CORE     9     Ti    Ti    0.0000     9
Ti    1.229069439     2.981079673     1.174206246     CORE     10    Ti    Ti    0.0000     10
Ti    3.782401731     0.815101600     0.622169350     CORE     11    Ti    Ti    0.0000     11
Ti    1.676014186     0.472891497     3.077286429     CORE     12    Ti    Ti    0.0000     12
end
end
```

Str-3 Energy from VASP is -106.79

```
!DATE
PBC     8.1377     5.5827     8.1097     77.2279     115.0775     77.5175
O     5.868971635     2.012104575     2.484667731     CORE     1     O     O     0.0000     1
O     7.945226094     5.276560441     0.686121521     CORE     2     O     O     0.0000     2
O     2.408985841     4.182532096     5.298365462     CORE     3     O     O     0.0000     3
O     6.969795475     4.623058661     3.426951470     CORE     4     O     O     0.0000     4
O     1.450988860     5.966791209     3.087043011     CORE     5     O     O     0.0000     5
O     3.804742005     6.732636391     4.837565122     CORE     6     O     O     0.0000     6
O     4.073939819     4.471017281     2.912760957     CORE     7     O     O     0.0000     7
O     5.055918829     2.948190089     5.156470215     CORE     8     O     O     0.0000     8
Ti    7.896030614     5.833088035     2.421635761     CORE     9     Ti    Ti    0.0000     9
Ti    2.908326187     5.376341016     4.007982538     CORE     10    Ti    Ti    0.0000     10
Ti    5.528888989     3.505460168     3.474252040     CORE     11    Ti    Ti    0.0000     11
Ti    3.339085169     2.676406857     5.752840050     CORE     12    Ti    Ti    0.0000     12
end
end
```

Str-4 Energy from VASP is -106.30

```
!DATE
PBC     3.4169     5.1875     8.6754     86.5497     101.3448     90.0459
O     1.076042399     1.141835544     6.709397986     CORE     1     O     O     0.0000     1
O     1.072230667     0.973182634     0.168346101     CORE     2     O     O     0.0000     2
O     -0.635863520    4.229521003     8.440496119     CORE     3     O     O     0.0000     3
O     1.074018404     2.126248253     2.831485910     CORE     4     O     O     0.0000     4
O     1.069697213     5.176622951     4.046253139     CORE     5     O     O     0.0000     5
O     -0.634037322    2.366902594     5.158369917     CORE     6     O     O     0.0000     6
O     1.072697533     3.593860196     6.927404914     CORE     7     O     O     0.0000     7
O     2.777727059     4.935909383     1.719574827     CORE     8     O     O     0.0000     8
Ti    -0.636038875    2.653687518     7.226786687     CORE     9     Ti    Ti    0.0000     9
Ti    1.073479671     0.424982420     2.216746229     CORE     10    Ti    Ti    0.0000     10
Ti    1.072823954     5.169858995     8.141136709     CORE     11    Ti    Ti    0.0000     11
Ti    1.074374377     1.690370849     4.660907849     CORE     12    Ti    Ti    0.0000     12
end
end
```

Str-5 Energy from VASP is -106.29

```
!DATE
PBC     8.0307     8.0256     5.8150     110.8864     69.0818     82.9311
O     3.591884064     1.911379118     2.172448537     CORE     1     O     O     0.0000     1
O     6.068317433     1.174807338     0.410385099     CORE     2     O     O     0.0000     2
O     8.924878934     3.132443563     2.367895761     CORE     3     O     O     0.0000     3
O     9.220231305     0.205872842     2.840501788     CORE     4     O     O     0.0000     4
O     8.040887783     5.960827269     2.547620878     CORE     5     O     O     0.0000     5
O     6.635925578     0.545533258     4.311507528     CORE     6     O     O     0.0000     6
O     6.058637220     3.780450726     1.884495975     CORE     7     O     O     0.0000     7
O     10.221167175    2.179108589     4.813112225     CORE     8     O     O     0.0000     8
Ti    1.988922690     1.844426812     3.034503581     CORE     9     Ti    Ti    0.0000     9
Ti    5.001858090     2.478630288     1.157684187     CORE     10    Ti    Ti    0.0000     10
```

Ti 7.782585370 4.373609951 1.692428650 CORE 11 Ti Ti 0.0000 11
Ti 7.793761871 -0.677949975 3.564450202 CORE 12 Ti Ti 0.0000 12
end
end

Str-6 Energy from VASP is -105.96
!DATE
PBC 3.1722 7.5314 6.0514 104.7018 105.1494 77.9269
O 1.983414512 0.354015447 0.678750650 CORE 1 O O 0.0000 1
O 0.390478552 4.325732397 5.242460023 CORE 2 O O 0.0000 2
O 1.979767882 1.581323454 4.160842142 CORE 3 O O 0.0000 3
O 1.975711568 2.929227058 0.249614211 CORE 4 O O 0.0000 4
O 0.390723125 1.725342645 2.238470063 CORE 5 O O 0.0000 5
O 1.974254360 6.514999894 2.667678506 CORE 6 O O 0.0000 6
O -1.186828335 -0.734684252 4.466161101 CORE 7 O O 0.0000 7
O 1.977183456 3.886057570 3.385642779 CORE 8 O O 0.0000 8
Ti 1.974708686 5.408297113 4.470116849 CORE 9 Ti Ti 0.0000 9
Ti 0.393336880 2.803540473 4.157750333 CORE 10 Ti Ti 0.0000 10
Ti 0.384928593 1.799976290 0.307226417 CORE 11 Ti Ti 0.0000 11
Ti 1.990539418 0.279140984 2.610300749 CORE 12 Ti Ti 0.0000 12
end
end

Str-7 Energy from VASP is -105.82
!DATE
PBC 2.9553 5.9700 9.3990 74.2814 89.9993 90.0006
O 0.791684930 7.615440174 8.610241779 CORE 1 O O 0.0000 1
O 0.791609498 3.553443003 3.929106915 CORE 2 O O 0.0000 2
O 2.269279723 5.755215887 2.760823043 CORE 3 O O 0.0000 3
O 0.791452990 7.283976966 5.001353159 CORE 4 O O 0.0000 4
O 2.269339579 3.678447097 1.377576459 CORE 5 O O 0.0000 5
O 2.269342529 4.007270431 9.008612214 CORE 6 O O 0.0000 6
O 2.269293666 3.123995044 6.574284777 CORE 7 O O 0.0000 7
O 0.791670891 5.445471125 7.581615779 CORE 8 O O 0.0000 8
Ti 2.269160985 2.402132863 4.869459463 CORE 9 Ti Ti 0.0000 9
Ti 0.791742354 3.422232939 7.972954274 CORE 10 Ti Ti 0.0000 10
Ti 0.791610701 4.682557242 2.482100854 CORE 11 Ti Ti 0.0000 11
Ti 2.269347594 6.139620602 8.599850851 CORE 12 Ti Ti 0.0000 12
end
end

Str-8 Energy from VASP is -105.65
!DATE
PBC 7.8704 7.5524 2.9162 89.4477 83.9347 107.5610
O 4.852963740 3.741068877 1.008911947 CORE 1 O O 0.0000 1
O 4.234893082 0.251018308 1.275088135 CORE 2 O O 0.0000 2
O 6.432399842 1.747424407 2.362241556 CORE 3 O O 0.0000 3
O 2.830709831 5.037127687 2.642726719 CORE 4 O O 0.0000 4
O -0.504853174 3.930222178 0.729971767 CORE 5 O O 0.0000 5
O 1.728457693 0.180006311 1.544180721 CORE 6 O O 0.0000 6
O 4.448837054 6.540980642 0.924996734 CORE 7 O O 0.0000 7
O 2.855866076 2.517891426 2.759485564 CORE 8 O O 0.0000 8
Ti 3.075327481 3.840485504 1.199371540 CORE 9 Ti Ti 0.0000 9
Ti 6.494366268 3.518367029 2.302938373 CORE 10 Ti Ti 0.0000 10
Ti 3.653969657 7.165554947 2.410480133 CORE 11 Ti Ti 0.0000 11
Ti 2.910818236 0.654561615 2.856968722 CORE 12 Ti Ti 0.0000 12
end
end

```
TiO2-OII        Energy from VASP is -104.21
!DATE
PBC   3.1747   6.3125   5.2386   90.1138   89.9999   90.0110
O    2.410624712   5.292048280   2.667179022   CORE   1   O   O   0.0000   1
O    0.824035880   2.129203698   5.156664289   CORE   2   O   O   0.0000   2
O    0.824557299   0.897448688   2.539497205   CORE   3   O   O   0.0000   3
O    2.411480559   4.061321129   0.056831716   CORE   4   O   O   0.0000   4
O    0.823564506   5.721239749   4.509014515   CORE   5   O   O   0.0000   5
O    2.411560690   2.570818515   3.319468222   CORE   6   O   O   0.0000   6
O    2.411729665   0.466341321   0.697486462   CORE   7   O   O   0.0000   7
O    0.823871852   3.616592051   1.893824964   CORE   8   O   O   0.0000   8
Ti   2.412113809   0.591082712   3.896715140   CORE   9   Ti  Ti  0.0000   9
Ti   0.824242527   3.745868410   3.932681043   CORE   10  Ti  Ti  0.0000   10
Ti   0.823111421   5.595303970   1.310487526   CORE   11  Ti  Ti  0.0000   11
Ti   2.411312641   2.440943172   1.279314338   CORE   12  Ti  Ti  0.0000   12
end
end

TiO2-OI        Energy from VASP is -212.57
!DATE
PBC   9.4352   4.9894   4.8433   90.0000   90.0000   90.0000
O    6.713616664   4.367977188   0.670449006   CORE   1   O   O   0.0000   1
O    4.951016256   1.185106423   2.403201071   CORE   2   O   O   0.0000   2
O    7.439169497   0.621467312   3.092106240   CORE   3   O   O   0.0000   3
O    9.201774917   3.804305446   4.824871544   CORE   4   O   O   0.0000   4
O    2.721542933   1.873264596   1.751265005   CORE   5   O   O   0.0000   5
O    4.484143627   3.679819340   0.018514634   CORE   6   O   O   0.0000   6
O    1.995993934   3.116180635   4.172941733   CORE   7   O   O   0.0000   7
O    0.233388044   1.309591557   2.440175599   CORE   8   O   O   0.0000   8
O    2.721544373   0.621435397   4.172932236   CORE   9   O   O   0.0000   9
O    4.484145555   3.804305925   2.440180227   CORE   10  O   O   0.0000   10
O    1.995991801   4.367945329   1.751274732   CORE   11  O   O   0.0000   11
O    0.233386381   1.185107458   0.018509614   CORE   12  O   O   0.0000   12
O    6.713618187   3.116148055   3.092116246   CORE   13  O   O   0.0000   13
O    4.951018241   1.309593457   4.824866763   CORE   14  O   O   0.0000   14
O    7.439167329   1.873232129   0.670439300   CORE   15  O   O   0.0000   15
O    9.201773205   3.679820626   2.403205695   CORE   16  O   O   0.0000   16
Ti   1.085403067   2.703701099   1.212470027   CORE   17  Ti  Ti  0.0000   17
Ti   3.632126790   2.285714790   3.634129317   CORE   18  Ti  Ti  0.0000   18
Ti   8.349757185   0.208987620   1.209245214   CORE   19  Ti  Ti  0.0000   19
Ti   5.803036438   4.780427178   3.630918364   CORE   20  Ti  Ti  0.0000   20
Ti   8.349758899   2.285711609   3.630911219   CORE   21  Ti  Ti  0.0000   21
Ti   5.803034034   2.703698102   1.209251901   CORE   22  Ti  Ti  0.0000   22
Ti   1.085404823   4.780424766   3.634136035   CORE   23  Ti  Ti  0.0000   23
Ti   3.632124309   0.208985105   1.212462930   CORE   24  Ti  Ti  0.0000   24
end
end

brookite        Energy from VASP is -429.98
!DATE
PBC   10.7943   5.1725   10.7892   89.9999   118.5747   90.0002
O    -2.875841735   3.602509630   5.348084071   CORE   1   O   O   0.0000   1
O    3.158796423    3.182974405   6.419112255   CORE   2   O   O   0.0000   2
O    -4.215074226   4.353236874   7.815537779   CORE   3   O   O   0.0000   3
O    -0.829921589   1.016098200   8.788134382   CORE   4   O   O   0.0000   4
O    7.750752288    5.018308903   0.708477931   CORE   5   O   O   0.0000   5
O    7.147244304    1.016121625   4.050375749   CORE   6   O   O   0.0000   6
```

O 0.349244915 2.432212477 6.415862105 CORE 7 O O 0.0000 7
O -2.542358525 1.016205180 6.599940841 CORE 8 O O 0.0000 8
O 3.761790011 4.353217513 3.078210836 CORE 9 O O 0.0000 9
O 4.263562617 3.182736349 8.968504928 CORE 10 O O 0.0000 10
O 1.446158320 3.182681086 4.231365665 CORE 11 O O 0.0000 11
O 5.027170269 5.018640549 5.532239736 CORE 12 O O 0.0000 12
O 2.209979308 5.018565393 0.795043011 CORE 13 O O 0.0000 13
O 4.338197434 1.766731192 4.047236609 CORE 14 O O 0.0000 14
O 2.217472475 0.596637278 5.528751628 CORE 15 O O 0.0000 15
O 5.434868938 1.016375836 1.862319200 CORE 16 O O 0.0000 16
O -1.771143608 3.602268900 7.897626298 CORE 17 O O 0.0000 17
O 8.326756463 2.432235317 1.678026805 CORE 18 O O 0.0000 18
O 5.603152946 2.432164998 6.501623918 CORE 19 O O 0.0000 19
O 5.101572079 3.602663355 0.610672981 CORE 20 O O 0.0000 20
O -3.638866570 1.766743497 8.784948191 CORE 21 O O 0.0000 21
O 10.194928179 0.596727937 0.790887413 CORE 22 O O 0.0000 22
O 9.014832315 4.353127597 3.162992236 CORE 23 O O 0.0000 23
O 3.929989888 0.596545484 7.716608522 CORE 24 O O 0.0000 24
O 1.037852835 4.353014562 7.900730949 CORE 25 O O 0.0000 25
O 1.614223300 1.767063394 8.869990338 CORE 26 O O 0.0000 26
O -1.203075104 1.767126545 4.132688683 CORE 27 O O 0.0000 27
O -0.226973028 5.018237874 5.446254238 CORE 28 O O 0.0000 28
O 0.341619083 3.183011510 1.681903723 CORE 29 O O 0.0000 29
O 2.785730985 2.432124510 1.764478852 CORE 30 O O 0.0000 30
O 1.112321211 0.596472371 2.979408037 CORE 31 O O 0.0000 31
O 6.205984518 3.602322603 3.159895673 CORE 32 O O 0.0000 32
Ti 9.430142606 4.114803320 1.364630443 CORE 33 Ti Ti 0.0000 33
Ti 4.563878546 2.670840119 2.259669122 CORE 34 Ti Ti 0.0000 34
Ti -2.535737684 0.084365403 8.471877754 CORE 35 Ti Ti 0.0000 35
Ti 5.441479626 0.084501179 3.733728341 CORE 36 Ti Ti 0.0000 36
Ti 3.392332004 1.528348276 9.365317407 CORE 37 Ti Ti 0.0000 37
Ti 1.983732344 4.114583943 2.582663974 CORE 38 Ti Ti 0.0000 38
Ti -0.065294441 2.670739032 8.214261317 CORE 39 Ti Ti 0.0000 39
Ti 1.106274367 1.528594997 1.108290090 CORE 40 Ti Ti 0.0000 40
Ti 1.452996335 4.114684278 6.102309735 CORE 41 Ti Ti 0.0000 41
Ti 7.911841666 2.670650138 3.476235527 CORE 42 Ti Ti 0.0000 42
Ti 0.574655329 1.528216453 4.628511943 CORE 43 Ti Ti 0.0000 43
Ti 4.801567099 4.114521240 7.319468161 CORE 44 Ti Ti 0.0000 44
Ti -2.005179451 0.084388176 4.950897703 CORE 45 Ti Ti 0.0000 45
Ti -3.412901056 2.670580066 6.997118548 CORE 46 Ti Ti 0.0000 46
Ti 3.923261657 1.528501955 5.845690514 CORE 47 Ti Ti 0.0000 47
Ti 5.972585246 0.084604998 0.213351313 CORE 48 Ti Ti 0.0000 48
end
end

phase-87 Energy from VASP is -429.95
!DATE
PBC 3.7505 15.5832 15.5604 94.0894 103.9334 76.0736
O 3.301163236 1.259446670 2.899229677 CORE 1 O O 0.0000 1
O 3.301276295 7.338823944 2.369563555 CORE 2 O O 0.0000 2
O 3.301795401 4.557046194 5.685874557 CORE 3 O O 0.0000 3
O 1.426716811 3.933814346 7.133418440 CORE 4 O O 0.0000 4
O 1.425523608 5.668962020 1.138626605 CORE 5 O O 0.0000 5
O 1.425731085 2.713930390 1.179993746 CORE 6 O O 0.0000 6
O 1.425732264 2.928891975 4.130380043 CORE 7 O O 0.0000 7
O 1.426294362 5.884212761 4.088889575 CORE 8 O O 0.0000 8
O 5.176408833 8.822017960 2.899229677 CORE 9 O O 0.0000 9
O 5.176521892 14.901395234 2.369563555 CORE 10 O O 0.0000 10

O	5.177040998	12.119617484	5.685874557	CORE	11	O	O	0.0000	11
O	3.301962408	11.496385636	7.133418440	CORE	12	O	O	0.0000	12
O	3.300769205	13.231533310	1.138626605	CORE	13	O	O	0.0000	13
O	3.300976682	10.276501680	1.179993746	CORE	14	O	O	0.0000	14
O	3.300977861	10.491463265	4.130380043	CORE	15	O	O	0.0000	15
O	3.301539959	13.446784051	4.088889575	CORE	16	O	O	0.0000	16
O	1.427738760	1.152357112	10.449747845	CORE	17	O	O	0.0000	17
O	1.427851819	7.231734386	9.920081723	CORE	18	O	O	0.0000	18
O	1.428370925	4.449956636	13.236392725	CORE	19	O	O	0.0000	19
O	-0.446707665	3.826724788	14.683936608	CORE	20	O	O	0.0000	20
O	-0.447900868	5.561872462	8.689144773	CORE	21	O	O	0.0000	21
O	-0.447693391	2.606840832	8.730511914	CORE	22	O	O	0.0000	22
O	-0.447692212	2.821802417	11.680898211	CORE	23	O	O	0.0000	23
O	-0.447130114	5.777123203	11.639407743	CORE	24	O	O	0.0000	24
O	3.302984358	8.714928401	10.449747845	CORE	25	O	O	0.0000	25
O	3.303097417	14.794305675	9.920081723	CORE	26	O	O	0.0000	26
O	3.303616523	12.012527925	13.236392725	CORE	27	O	O	0.0000	27
O	1.428537933	11.389296077	14.683936608	CORE	28	O	O	0.0000	28
O	1.427344730	13.124443751	8.689144773	CORE	29	O	O	0.0000	29
O	1.427552207	10.169412121	8.730511914	CORE	30	O	O	0.0000	30
O	1.427553386	10.384373706	11.680898211	CORE	31	O	O	0.0000	31
O	1.428115484	13.339694492	11.639407743	CORE	32	O	O	0.0000	32
Ti	1.426420275	4.370020276	5.176563333	CORE	33	Ti	Ti	0.0000	33
Ti	1.426091992	6.842279153	2.586646288	CORE	34	Ti	Ti	0.0000	34
Ti	1.424945584	4.228027082	0.092251333	CORE	35	Ti	Ti	0.0000	35
Ti	1.425856300	1.755850795	2.682191476	CORE	36	Ti	Ti	0.0000	36
Ti	3.301665872	11.932591566	5.176563333	CORE	37	Ti	Ti	0.0000	37
Ti	3.301337589	14.404850443	2.586646288	CORE	38	Ti	Ti	0.0000	38
Ti	3.300191181	11.790598372	0.092251333	CORE	39	Ti	Ti	0.0000	39
Ti	3.301101897	9.318422085	2.682191476	CORE	40	Ti	Ti	0.0000	40
Ti	-0.447004201	4.262930718	12.727081501	CORE	41	Ti	Ti	0.0000	41
Ti	-0.447332484	6.735189595	10.137164456	CORE	42	Ti	Ti	0.0000	42
Ti	-0.448478892	4.120937524	7.642769501	CORE	43	Ti	Ti	0.0000	43
Ti	-0.447568176	1.648761237	10.232709644	CORE	44	Ti	Ti	0.0000	44
Ti	1.428241397	11.825502007	12.727081501	CORE	45	Ti	Ti	0.0000	45
Ti	1.427913114	14.297760884	10.137164456	CORE	46	Ti	Ti	0.0000	46
Ti	1.426766706	11.683508813	7.642769501	CORE	47	Ti	Ti	0.0000	47
Ti	1.427677422	9.211332526	10.232709644	CORE	48	Ti	Ti	0.0000	48

end
end

phase-139 Energy from VASP is -429.25
!DATE
PBC 11.1993 7.5881 11.1803 99.7705 88.4122 99.7535

O	6.077922890	2.803009270	0.972590490	CORE	1	O	O	0.0000	1
O	-0.464606670	5.528084860	6.942270980	CORE	2	O	O	0.0000	2
O	2.459517920	6.030872400	7.614437460	CORE	3	O	O	0.0000	3
O	6.104394510	6.656954600	3.935821890	CORE	4	O	O	0.0000	4
O	0.178186070	1.788869780	6.942153120	CORE	5	O	O	0.0000	5
O	5.615127420	-1.126237140	10.207240810	CORE	6	O	O	0.0000	6
O	1.293664470	1.980742830	10.244170920	CORE	7	O	O	0.0000	7
O	4.630168500	4.478986900	8.689008530	CORE	8	O	O	0.0000	8
O	0.650905370	5.720025120	10.244162900	CORE	9	O	O	0.0000	9
O	6.747128940	2.917784590	3.935789770	CORE	10	O	O	0.0000	10
O	8.119048280	1.228887370	5.819181630	CORE	11	O	O	0.0000	11
O	3.102309860	2.291685310	7.614416540	CORE	12	O	O	0.0000	12
O	7.476264760	4.968148650	5.819233710	CORE	13	O	O	0.0000	13
O	8.161297580	1.236242410	2.076068740	CORE	14	O	O	0.0000	14

O	5.435138610	6.542199870	0.972562990	CORE	15	O	O	0.0000	15
O	1.593073970	0.107361620	8.726133370	CORE	16	O	O	0.0000	16
O	2.511114960	6.039797480	0.301355960	CORE	17	O	O	0.0000	17
O	9.937094120	-0.383388890	5.837913900	CORE	18	O	O	0.0000	18
O	10.066293800	5.413435430	3.978887860	CORE	19	O	O	0.0000	19
O	4.972390110	2.613011070	10.207207470	CORE	20	O	O	0.0000	20
O	7.518595730	4.975418680	2.076043250	CORE	21	O	O	0.0000	21
O	8.693845160	7.102329910	2.096215840	CORE	22	O	O	0.0000	22
O	6.656954300	-0.947149290	6.885787320	CORE	23	O	O	0.0000	23
O	5.272874860	0.739833990	8.688996640	CORE	24	O	O	0.0000	24
O	9.294383970	3.355767270	5.837900330	CORE	25	O	O	0.0000	25
O	-0.401175440	5.539234510	1.028702280	CORE	26	O	O	0.0000	26
O	0.241593750	1.800035390	1.028570000	CORE	27	O	O	0.0000	27
O	6.014190290	2.792022990	6.885862230	CORE	28	O	O	0.0000	28
O	9.336560310	3.363198700	2.096216730	CORE	29	O	O	0.0000	29
O	3.153816220	2.300596800	0.301371360	CORE	30	O	O	0.0000	30
O	0.950311810	3.846588300	8.726095360	CORE	31	O	O	0.0000	31
O	10.709082760	1.674228930	3.978898780	CORE	32	O	O	0.0000	32
Ti	9.474342310	5.311717500	2.258238810	CORE	33	Ti	Ti	0.0000	33
Ti	10.079268550	1.565835970	5.684974360	CORE	34	Ti	Ti	0.0000	34
Ti	1.770151710	0.137898300	10.710602680	CORE	35	Ti	Ti	0.0000	35
Ti	0.778852070	5.741931740	8.255652100	CORE	36	Ti	Ti	0.0000	36
Ti	1.127455250	3.877101980	10.710597330	CORE	37	Ti	Ti	0.0000	37
Ti	6.733717470	6.765300950	2.229499790	CORE	38	Ti	Ti	0.0000	38
Ti	1.421599720	2.002736320	8.255627760	CORE	39	Ti	Ti	0.0000	39
Ti	9.436496190	5.305033530	5.685038490	CORE	40	Ti	Ti	0.0000	40
Ti	10.117068730	1.572505780	2.258258810	CORE	41	Ti	Ti	0.0000	41
Ti	5.144046100	0.717690910	10.677666460	CORE	42	Ti	Ti	0.0000	42
Ti	7.338554770	3.019556600	5.656680890	CORE	43	Ti	Ti	0.0000	43
Ti	4.501268540	4.456903090	10.677672760	CORE	44	Ti	Ti	0.0000	44
Ti	5.438660070	-1.156533490	8.222609290	CORE	45	Ti	Ti	0.0000	45
Ti	7.981277490	-0.719645150	5.656695610	CORE	46	Ti	Ti	0.0000	46
Ti	4.795961520	2.582677790	8.222597580	CORE	47	Ti	Ti	0.0000	47
Ti	7.376466830	3.026088080	2.229502730	CORE	48	Ti	Ti	0.0000	48

end
end

IS (phase-87) of pathway between phase-87 to anatase Energy from VASP is -107.48
!DATE
PBC 3.7347 7.8225 7.7649 94.5674 103.9351 76.1829

O	3.285903140	1.234500890	2.919231410	CORE	1	O	O	0.0000	1
O	3.286738670	7.353537630	2.338462710	CORE	2	O	O	0.0000	2
O	3.284469530	4.567030680	5.688790040	CORE	3	O	O	0.0000	3
O	1.417329970	3.843997050	7.103139280	CORE	4	O	O	0.0000	4
O	1.419599090	5.637960560	1.163501350	CORE	5	O	O	0.0000	5
O	1.419419960	2.684431140	1.163426780	CORE	6	O	O	0.0000	6
O	1.417756340	2.949995190	4.094109870	CORE	7	O	O	0.0000	7
O	1.418368710	5.903270370	4.093976730	CORE	8	O	O	0.0000	8
Ti	1.416798560	4.369748510	5.164059450	CORE	9	Ti	Ti	0.0000	9
Ti	1.419353600	6.847812580	2.586676040	CORE	10	Ti	Ti	0.0000	10
Ti	1.420208730	4.218084290	0.093592320	CORE	11	Ti	Ti	0.0000	11
Ti	1.418628190	1.740178950	2.670813760	CORE	12	Ti	Ti	0.0000	12

end
end

FS (anatase) of pathway between phase-87 to anatase Energy from VASP is -107.62
!DATE
PBC 3.8035 7.7325 5.5562 112.3479 110.0077 75.7697

O	3.347004150	0.471511740	1.119754860	CORE	1	O	O	0.0000	1

O	1.445474330	-0.555893800	2.324828440	CORE	2	O	O	0.0000	2
O	3.346986970	3.368631000	3.587193420	CORE	3	O	O	0.0000	3
O	1.445429830	2.345394590	4.795858920	CORE	4	O	O	0.0000	4
O	1.444934360	4.943147220	1.737292520	CORE	5	O	O	0.0000	5
O	-0.456077130	0.346832350	4.205188400	CORE	6	O	O	0.0000	6
O	1.444964430	2.467686990	1.708032810	CORE	7	O	O	0.0000	7
O	1.444822080	5.367130390	4.177144380	CORE	8	O	O	0.0000	8
Ti	1.445072490	3.642388430	3.263498930	CORE	9	Ti	Ti	0.0000	9
Ti	-0.455947940	-0.827873940	2.650748680	CORE	10	Ti	Ti	0.0000	10
Ti	1.445413030	3.768665230	0.182333090	CORE	11	Ti	Ti	0.0000	11
Ti	1.445364800	0.743729020	0.794478750	CORE	12	Ti	Ti	0.0000	12

end
end

TS of pathway between phase-87 to anatase Energy from VASP is -106.74

!DATE

PBC 3.7976 8.0543 6.0104 104.4287 108.4121 76.3682

O	3.341917558	0.910533440	1.569423090	CORE	1	O	O	0.0000	1
O	1.443324878	-0.377184201	2.509462438	CORE	2	O	O	0.0000	2
O	3.341591052	3.746384816	4.051733856	CORE	3	O	O	0.0000	3
O	1.443027640	3.146012868	5.504025290	CORE	4	O	O	0.0000	4
O	1.443245052	5.568388539	1.368788185	CORE	5	O	O	0.0000	5
O	-0.455304603	1.043822888	5.412711344	CORE	6	O	O	0.0000	6
O	1.442797161	3.073205238	2.150150228	CORE	7	O	O	0.0000	7
O	1.443243772	5.883803145	4.063042030	CORE	8	O	O	0.0000	8
Ti	1.442807948	4.110225891	3.823426058	CORE	9	Ti	Ti	0.0000	9
Ti	1.443182014	6.922422129	2.483566700	CORE	10	Ti	Ti	0.0000	10
Ti	1.442857057	3.962878163	0.247236248	CORE	11	Ti	Ti	0.0000	11
Ti	1.443006896	1.354981002	1.459212569	CORE	12	Ti	Ti	0.0000	12

end
end
